# Cellular basis of HFpEF: contributions of abnormal lipids

**DOI:** 10.3389/fcvm.2026.1885850

**Published:** 2026-08-04

**Authors:** Huan-Hsing Chiang, Omer Akyol, Ming-Hsien Tsai, Camila Hochman-Mendez, Abdelmotagaly Elgalad, Darren Woodside, Chu-Huang Chen, Deepak L. Bhatt

**Affiliations:** 1 Molecular Cardiology Research Laboratories, Vascular and Medicinal Research, The Texas Heart Institute at Baylor College of Medicine, Houston, TX, United States; 2 School of Medicine, Texas Tech University Health Sciences Center, Lubbock, TX, United States; 3 Department of Child-Care, College of Humanities and Social Sciences, National Pingtung University of Science and Technology, Pingtung, Taiwan; 4 Regenerative Medicine Research Department, The Texas Heart Institute at Baylor College of Medicine, Houston, TX, United States; 5 Center for Preclinical Surgical & Interventional Research, The Texas Heart Institute at Baylor College of Medicine, Houston, TX, United States; 6 Molecular Cardiology Research Laboratories, The Texas Heart Institute at Baylor College of Medicine, Houston, TX, United States; 7 Mount Sinai Fuster Heart Hospital, Icahn School of Medicine at Mount Sinai, New York, NY, United States

**Keywords:** electronegative lipoproteins, HFpEF, LDL, lipids, metabolic dysfunction, VLDL

## Abstract

Heart failure with preserved ejection fraction (HFpEF), one of the largest unmet medical needs, is characterized by high morbidity and mortality and a lack of effective treatments. Numerous studies suggest that the potential contribution of dysfunctional metabolism to HFpEF, emphasizing the possible role of lipids, especially lipoproteins, in the disease's initiation and progression. Changes in circulating plasma lipids appear to play a role in mediating the pathophysiological processes that may contribute to the development of HFpEF through an imbalance in the circulating lipid supply, cardiac fat uptake, and fatty acid metabolism. This metabolic mismatch results in the accumulation of harmful lipid intermediates within the myocardium. This process, known as lipotoxicity, contributes to diastolic dysfunction. Furthermore, altered plasma lipid levels can potentiate systemic inflammatory responses, endothelial dysfunction, and myocardial fibrosis. Understanding the impact of lipid metabolism on these underlying mechanisms is emerging as a novel avenue for preventing and treating HFpEF. The intricate relationship between HFpEF and lipid metabolism may involve multifaceted factors, including insulin sensitivity and adipose tissue inflammation. Here, we have comprehensively analysed the evidence-based pathophysiological impact of broader lipid abnormalities [including very low-density lipoprotein (VLDL) and low-density lipoprotein] on HFpEF development. Furthermore, drawing on foundational studies in related metabolic disorders, we propose a novel hypothesis regarding the potential, yet underexplored role of electronegative VLDL (V5), a particularly harmful subfraction of VLDL, as a future therapeutic target in HFpEF.

## 1 Introduction

The clinical concept of heart failure with preserved ejection fraction (HFpEF) was first clearly and comprehensively defined in 1982 by Dr. Luchi and colleagues, who characterized a cohort of patients presenting with heart failure symptoms despite having a preserved left ventricular ejection fraction (1). According to the European Society of Cardiology's stepwise diagnostic algorithm, a diagnosis of HFpEF requires the simultaneous presence of typical heart failure symptoms, a preserved ejection fraction of 50% or greater, and objective evidence of underlying structural or functional cardiac abnormalities, such as diastolic dysfunction or elevated natriuretic peptide levels (2).

Indeed, the cardiac manifestation of HFpEF is now understood to be part of a broader, pro-inflammatory multisystemic syndrome driven by various risk factors and clinical phenotypes (3). The presentation of HFpEF differs depending on a patient's underlying comorbidities; for example, a significant subset of patients develops the disease driven primarily by metabolic disorders, such as obesity (4) and elevated low-density lipoprotein cholesterol (LDL-C) (5). Crucially, these elevations in circulating lipids can directly provoke systemic and coronary microvascular inflammation, which subsequently plays a central role in driving the pathogenesis of HFpEF. Alongside fueling this inflammatory cascade, alterations in circulating plasma lipids further mediate HFpEF development through direct structural and metabolic disruptions. These processes likely involve a mismatch among circulating lipid supply, cardiac fat uptake, and fatty acid metabolism, leading to the myocardial accumulation of toxic lipid intermediates that result in diastolic dysfunction. Additionally, altered plasma lipid levels can directly disrupt cardiac metabolism by reducing cardiac metabolic flexibility, depleting cardiac adenosine triphosphate (ATP), and impairing cardiac function (6). Furthermore, circulating lipids affect the phospholipid composition of cell membranes, thereby altering cardiomyocyte function and impairing left ventricular diastolic performance (7).

Despite the growing prevalence of HFpEF and its severe impact on patient quality of life, effective targeted therapies are limited. Therefore, updated comprehensive analyses of the circulating lipid profile in patients with HFpEF may improve our understanding of relevant circulating lipid species and corresponding molecular mechanisms in the heart. This review aims to explore major topics associated with HFpEF by discussing the latest evidence on broad lipid abnormalities, including highly established cellular drivers such as ceramides, sphingolipids, and myocardial stenosis. Although the evidence indicating the role of these downstream intracellular lipid intermediates in myocardial injury is robust, there is a critical knowledge gap regarding the specific upstream circulating lipoproteins that initiate this tissue-level lipotoxicity. Thus, we introduce a hypothesis-driven perspective on the role of electronegative very low-density lipoprotein (VLDL) in this process; specifically, V5, a novel, highly pathogenic systemic trigger, may bridge the gap between circulating dyslipidemia and intracellular lipid accumulation. Direct clinical studies on V5 in HFpEF populations are currently limited; however, here, we extrapolate from its established inflammatory and lipotoxic effects in other metabolic diseases to propose V5 as a potential, previously unrecognized contributor to HFpEF pathogenesis. Using this dual approach of systematically reviewing robustly evidenced lipid classes while generating an upstream hypothesis, we aim to provide a new perspective to improve clinical and therapeutic management of HFpEF.

Furthermore, it is increasingly recognized that HFpEF is not a singular disease, but rather a highly heterogeneous clinical syndrome comprising several distinct phenotypes, including age-related, hypertensive, atrial fibrillation-predominant, and obese/cardiometabolic variants (8, 9). While traditional models often treated HFpEF uniformly, contemporary approaches emphasize that the underlying cellular drivers vary significantly among these subgroups. The lipid abnormalities detailed in this review are central to the pathogenesis of the obese/cardiometabolic HFpEF phenotype, which is primarily driven by systemic metabolic dysfunction, insulin resistance, and visceral adiposity, creating a unique environment of systemic lipid oversupply (10, 11). Consequently, the mechanisms of myocardial steatosis, the accumulation of toxic lipid intermediates, such as ceramides and diacylglycerols (DAGs), and subsequent lipotoxicity are most profoundly expressed in these patients. Therefore, although lipid derangements may intersect with other risk factors such as aging and hypertension, the targeted evaluation of circulating lipid species and the lipotoxic mechanisms discussed herein are most critically relevant to the cardiometabolic HFpEF population.

## 2 Contemporary cellular mechanisms underlying HFpEF pathophysiology

HFpEF is a complex disorder characterized by intricate pathophysiology, including oxidative stress and inflammation. Oxidative stress arises from an imbalance in cellular redox states, characterized by either heightened production of reactive oxygen species (ROS) or reduced availability of antioxidants. Although ROS play essential roles in cellular signaling pathways, excessive levels can cause significant cellular and myocardial damage. They can impair contractile function by modifying key proteins involved in excitation-contraction coupling. Additionally, ROS activate hypertrophic signaling kinases and stimulate cardiac fibroblast proliferation, leading to the activation of matrix metalloproteinases and subsequent extracellular matrix (ECM) remodeling (12, 13). Furthermore, ROS induce direct post-translational modifications of cellular proteins, such as the creation of disulfide bonds, glutathionylation, and nitrosylation, that subsequently alter target protein function (14, 15). These processes trigger signaling cascades that affect endothelial cells, cardiomyocytes, and the ECM, contributing to the progression of HFpEF (16).

The “inflammatory coronary microvascular dysfunction” hypothesis proposes a central mechanism of HFpEF, suggesting that comorbidity-driven systemic inflammation triggers endothelial dysfunction, which subsequently leads to cardiac remodeling. In 2013, Paulus and Tschope proposed that comorbidities like diabetes, hypertension, aging, and obesity drive a systemic pro-inflammatory state in HFpEF (17). This inflammation leads to increased levels of circulating cytokines, which trigger oxidative enzymes within the endothelium, ultimately reducing nitric oxide (NO) bioavailability. As a result, the NO-cGMP-protein kinase G (PKG) signaling pathway in neighboring cardiomyocytes is disrupted in the coronary microcirculation (18). Endothelial dysfunction leads to reduced NO bioavailability, impaired vasodilation, and increased vascular stiffness, all of which contribute to the overall cardiac dysfunction observed in HFpEF patients (19).

Cardiomyocyte alterations are significant in HFpEF. Unlike in heart failure with reduced ejection fraction (HFrEF), HFpEF is characterized by increased cytosolic calcium concentration, particularly through enhanced L-type calcium channel current. Active relaxation of cardiomyocytes is compromised in HFpEF, affecting the early diastolic phase and increasing passive stiffness (16, 20). Brutsaert et al. discovered that the NO-sGC-cGMP pathway affects not only vascular smooth muscle cells but also cardiomyocytes (21). Within the myocardium, cGMP activates PKG to act as a regulatory checkpoint on signaling pathways governing cardiomyocyte stiffness and hypertrophy (22). By affecting active relaxation and passive stiffness of the heart, endothelium-derived NO directly regulates cardiac diastolic function (23, 24).

ECM remodeling is another crucial aspect of HFpEF pathophysiology. Increased extracellular fibrosis has been shown in cardiac biopsies of HFpEF patients (25). This chronic, diffuse interstitial stiffening differs from the localized replacement scarring seen after acute ischemic injury. For example, forensic post-mortem studies of acute myocardial infarction demonstrate that the presence of pre-existing myocardial scar tissue and hypertrophy does not correlate with catastrophic structural failures such as ventricular wall rupture; rather, the acute ischemic insult itself is the primary driver (26). In contrast, the non-ischemic pathology of HFpEF involves a continuous, metabolically driven remodeling of the ECM. Under normal conditions, cardiac fibroblasts remain inactive within the ECM. However, when stimulated by inflammatory cytokines, they transform into myofibroblasts, which actively produce and secrete collagen (27, 28). Increased collagen deposition and cross-linking result in myocardial fibrosis, while changes in ECM composition and organization disrupt overall cardiac structure and function (16, 29, 30). Cardiac fibrosis, a well-documented complication in patients with chronic diabetes mellitus, is significantly driven by high blood glucose levels (31). In an *in vitro* study, cardiac fibroblasts cultured in high-glucose conditions produced excessive ECM proteins, including collagens, fibronectin, and matricellular macromolecules (32). These changes contribute significantly to the increased myocardial stiffness observed in HFpEF.

## 3 Understanding myocardial metabolism: the importance of lipids

**Figure 1:**
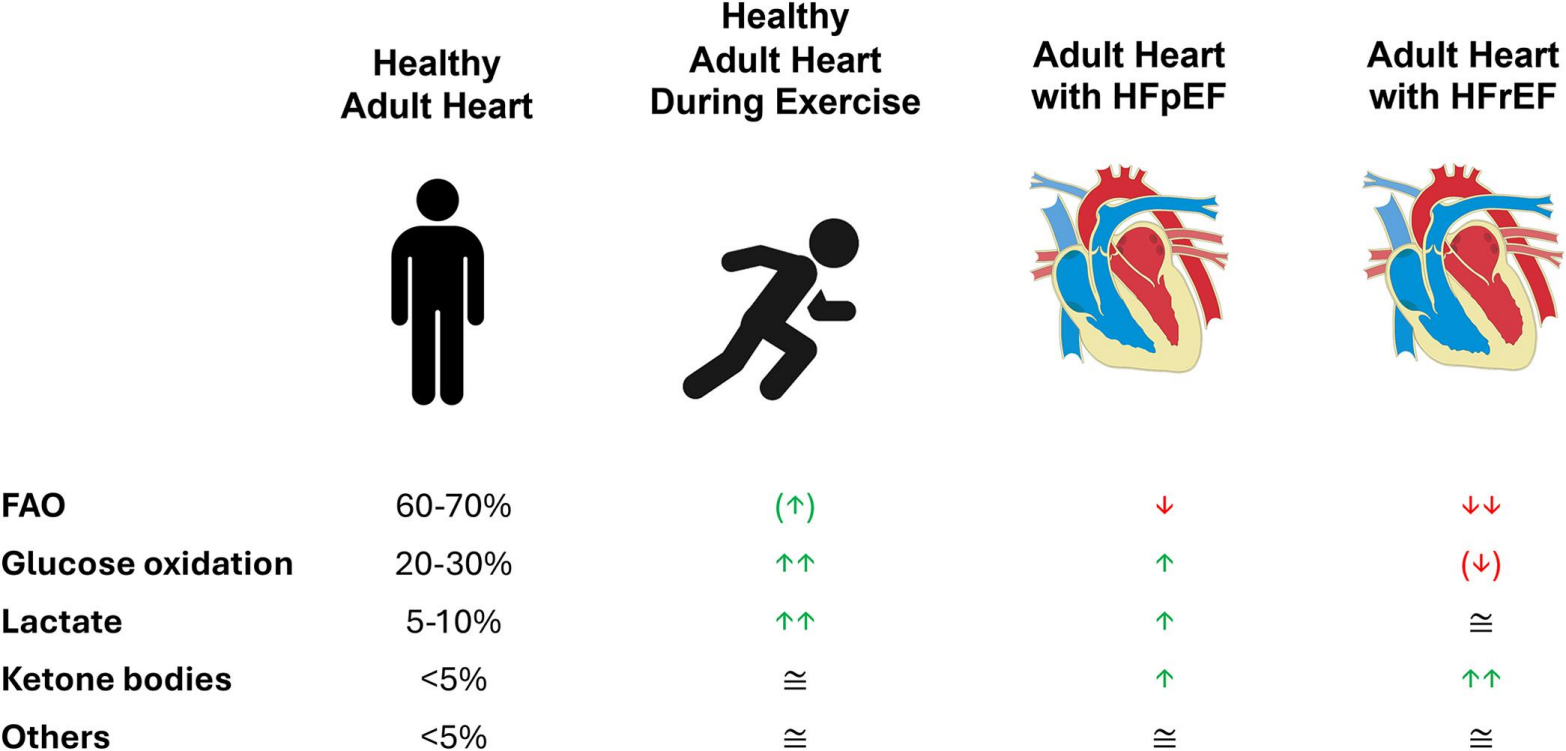
Established metabolic shifts in the heart. Cardiac metabolism in a healthy adult heart, a healthy heart during exercise, an adult heart with HFpEF, and an adult heart with HFrEF. A healthy adult heart relies primarily on fatty acid oxidation (FAO) for energy but maintains metabolic flexibility. For example, during exercise and increased demand, the heart seamlessly shifts to utilize more glucose and lactate. In HFpEF, cardiac hypertrophy along with mitochondrial inefficiency causes a decrease in FAO and a shift in substrate use. In contrast, the heart with HFrEF shows marked increase in the use of ketone bodies; the energy-deficient heart has the heaviest reliance on alternative fuels. All arrows are relative to metabolism in a healthy adult heart. Parentheses indicate a slight or variable change.

Myocardial metabolism is a complex and dynamic process that plays a crucial role in maintaining cardiac function. The heart is a high energy-demanding organ, and a primary fuel source for energy production in cardiomyocytes is lipids, which also serve as potential mediators of cardiac dysfunction. The intricate balance between lipid uptake, oxidation, and storage is crucial for maintaining cardiac health. Therefore, understanding the intricacies of lipid metabolism in the myocardium is necessary to elucidate the pathophysiology of various cardiac disorders, including HFpEF and lipotoxic cardiomyopathy (Figure 1).

In the healthy adult heart, fatty acid oxidation (FAO) accounts for approximately 60%–70% of ATP production, with glucose oxidation providing the remainder (33). Mitochondrial oxidative metabolism generates 95% of the heart's total energy, with the remaining 5% produced through anaerobic glycolysis (34). The heart obtains fatty acids from primarily three sources: non-esterified fatty acids, chylomicrons, and VLDL. The uptake of these lipids into cardiomyocytes is facilitated by specialized transporters, with CD36 playing a pivotal role in fatty acid translocation across the sarcolemma (33). Once inside the cardiomyocyte, fatty acids are activated to acyl-CoA esters and can either undergo β-oxidation in the mitochondria for energy production or be esterified to form triacylglycerols (TAGs) for storage in lipid droplets. The balance between lipid uptake, oxidation, and storage is tightly regulated to prevent lipotoxicity. However, perturbations in this homeostasis can contribute to the accumulation of lipids, inducing endoplasmic reticulum stress, mitochondrial dysfunction, and oxidative stress, all of which contribute to cardiomyocyte injury and death (35).

In pathological conditions such as obesity, diabetes, and heart failure, there is often a shift in myocardial substrate utilization from fatty acids to glucose. This metabolic remodeling is associated with a loss in both metabolic flexibility and optimal ATP production. Due to reduced FAO, lipid accumulation in the myocardium is frequently seen in these conditions (36). This phenomenon is attributed to a mismatch between lipid uptake and utilization, leading to the buildup of intracellular lipids. Excess lipids can be converted to toxic intermediates such as DAGs, ceramides, and acylcarnitines, which activate protein kinase C (PKC) isoforms and promote insulin resistance (35). The combination of decreased ATP production, low oxygen availability, and reduced ability to switch between energy substrates contributes to cardiac energetic dysfunction in HFpEF (37).

The regulation of myocardial lipid metabolism involves a complex interplay of transcription factors and signaling pathways. Key regulators include peroxisome proliferator-activated receptors (PPARs), particularly PPAR-α, which promotes the myocardial expression of genes involved in FAO. Additionally, the sterol regulatory element-binding protein (SREBP) family, especially the SREBP-1c isoform, acts as a master transcriptional regulator of *de novo* lipogenesis in the heart. In cardiomyocytes, SREBP-1c is strongly associated with the synthesis of lipids by directly upregulating the expression of crucial lipogenic enzymes, such as fatty acid synthase and acetyl-CoA carboxylase. Under pathological conditions, the maladaptive overactivation of SREBP-1c leads to excessive intramyocardial lipid biosynthesis and storage, directly contributing to cardiac steatosis and subsequent lipotoxicity (36, 38–41). Emerging evidence suggests that microRNAs also play a significant role in modulating cardiac lipid metabolism. These small non-coding RNAs can regulate the expression of genes involved in lipid uptake, oxidation, and storage, thereby affecting myocardial lipid homeostasis (19).

The lipotoxic mechanisms driving HFpEF are most fully appreciated by distinguishing the metabolic profile of HFpEF from that of HFrEF. The two phenotypes exhibit fundamentally divergent alterations in myocardial lipid handling. In HFrEF, the failing heart undergoes a metabolic shift resembling a “fetal gene program,” characterized by a marked downregulation of FAO and a compensatory reliance on glycolysis (42). This results in an “energy-starved” myocardium where lipids are underutilized. Conversely, HFpEF, frequently occurring in the context of systemic metabolic syndrome and obesity, is characterized by a state of lipid oversupply. In HFpEF, myocardial lipid uptake exceeds the oxidative capacity of mitochondria. This mismatch forces the diversion of excess fatty acids into non-oxidative pathways, leading to the intracellular accumulation of highly bioactive, toxic lipid intermediates such as ceramides and DAGs (43).

In line with these distinct metabolic profiles, proton (^1^H) magnetic resonance spectroscopy of the heart has shown that HFpEF patients have significantly more intramyocardial fat than do HFrEF or control subjects; this myocardial steatosis promotes lipotoxicity and may exacerbate diastolic stiffness and concentric remodeling, correlating with the severity of diastolic dysfunction independent of other risk factors (43). This clinical finding indicates the pathological role of lipids in HFpEF. Rather than simply causing an energy deficit, this lipid accumulation acts as an active signaling trigger that provokes mitochondrial alterations, reduces FAO, exacerbates ROS production, and impairs myocardial energetics Targeting these specific lipid metabolism pathways may be a critical therapeutic frontier for treating HFpEF (19).

## 4 Lipid dysregulation and abnormal lipids in HFpEF pathophysiology

Systemic dyslipidemia is frequently cited as a key metabolic determinant in HFpEF pathogenesis, but the clinical evidence linking traditional lipid parameters to adverse HFpEF outcomes remains surprisingly inconsistent. Notably, several observational studies suggest that standard circulating LDL-C is not a major determinant of HFpEF hospitalization or mortality, indicating that HFpEF prognosis is driven more by comorbidity burden and non-lipid mechanisms (44). This clinical discrepancy underscores a crucial pathophysiological distinction: the concentration of circulating plasma lipids does not directly reflect the severity of lipid accumulation within the myocardium.

In the context of HFpEF, lipotoxicity is fundamentally determined by systemic metabolic dysfunction rather than absolute plasma lipid concentrations. Severe insulin resistance and visceral adiposity can disrupt cardiac fuel handling by increasing fatty acid uptake relative to mitochondrial FAO, thereby promoting myocardial lipid accumulation and metabolic dysfunction (45). This localized metabolic mismatch forces the pathological intracellular accumulation of specific bioactive lipid intermediates, which may play crucial roles in the pathophysiology of HFpEF through multiple mechanisms (46, 47). Research has highlighted the varying pathological roles of the accumulation of these distinct lipid species in cardiac function. For example, cardiolipin, a unique phospholipid found in the inner mitochondrial membrane, is crucial for maintaining mitochondrial structure and function. Alterations in cardiolipin content or composition have been implicated in cardiac pathologies such as heart failure and ischemia-reperfusion injury (48). Furthermore, certain lipid classes (i.e., palmitic acid compared to oleic acid) incorporate more poorly into TAGs, thus initiating cell apoptosis (49). Several explanations have been suggested for the toxicity of palmitic acid, including changes in membrane flexibility, increased ceramide production, and elevated ROS levels (50, 51). Supporting this, a case-control study showed that two specific lipid metabolites were significantly associated with heart failure risk: ceramides and phosphatidylcholines (52). These findings support those of previous studies indicating that long-chain ceramides induce mitochondrial dysfunction and cell death in cardiomyocytes (53). Together, these studies underscore how lipid species are metabolized differently, and how their intracellular accumulation negatively affects endothelial function, cardiac fibrosis, and inflammatory responses, all of which are crucial components in understanding the HFpEF pathophysiology (Table 1).

**Table 1:** Lipid classes, metabolic characteristics, and their differential contributions to endothelial dysfunction, cardiac fibrosis, and inflammation in HFpEF pathophysiology.

Lipid class	Key metabolic features	Endothelial function	Cardiac fibrosis/structural remodeling	Inflammatory responses/HFpEF relevance	Level of Evidence
Triacylglycerols (TAGs)	Stored in adipose/ectopic sites; elevated in insulin resistance and obesity; reflected by TyG index.	Hypertriglyceridemia impairs NO bioavailability and microvascular function, especially in metabolic syndrome.	Promote myocardial steatosis/ lipotoxicity, driving diastolic stiffness and concentric remodeling in metabolic HFpEF.	Excess TAGs/FFAs drive low-grade inflammation; higher HFpEF risk with elevated TyG index.	Mechanistic, animal, observational human, and clinical studies (TyG indices)
Free fatty acids (FFA)	Mobilized from adipose tissue; β-oxidation in heart and skeletal muscle; oxidation in heart and skeletal muscle; oversupply in obesity/diabetes.	FFA overload induces endothelial oxidative stress and reduces NO, promoting coronary microvascular dysfunction.	Lipotoxic FFA metabolites cause cardiomyocyte apoptosis and interstitial fibrosis, worsening diastolic function.	Activate TLR and NF-κB pathways, enhancing cytokine production in metabolic HFpEF phenotypes.	Mechanistic, preclinical, observational human, and clinical studies
Cholesteryl esters (CE)	Packaged in LDL and HDL; remodeled by CETP and LCAT; altered CE species track with HFpEF severity.	Atherogenic CE-rich lipoproteins impair endothelial function and promote coronary microvascular rarefaction.	CE accumulation in vascular and cardiac macrophages contributes indirectly to fibrotic signaling.	Specific CE species correlate with inflammatory biomarkers and adverse clinical parameters in HFpEF.	Mechanistic, animal, observational human, and clinical studies
Phospholipids (PC, PE, LPC, cardiolipin)	Structural membrane lipids; reservoirs for bioactive mediators. Cardiolipin is unique to the inner mitochondrial membrane.	Altered phosphatidylcholine and lysophospholipid species associated with endothelial dysfunction and impaired vasodilation.	Imbalanced PC/PE ratios and cardiolipin depletion affect mitochondrial function and may favor profibrotic signaling.	Certain lysophospholipids are proinflammatory chemoattractants, while others can be neutral or anti-inflammatory. Mitochondrial debris from phospholipid breakdown acts as an inflammatory trigger.	Mechanistic, animal, observational human, and clinical studies
Sphingolipids (ceramides, sphingomyelins)	Central regulators of cell stress signaling; generated *de novo* or via sphingomyelin hydrolysis; elevated in cardiometabolic disease.	Ceramide accumulation in endothelium causes eNOS uncoupling, oxidative stress, and impaired vasorelaxation.	Ceramides drive cardiac fibrosis/remodeling; lowering them improves ventricular structure/function in heart failure models.	Strongly pro-inflammatory, amplifying cytokine production and leukocyte recruitment; recognized as key lipotoxic mediators in HFpEF.	Mechanistic, preclinical (animal), observational human, and clinical studies
Oxidized lipids (oxLDL, oxidized phospholipids)	Generated under oxidative stress; bind scavenger receptors and pattern recognition receptors.	Potently impair endothelial function, increase adhesion molecule expression, and promote microvascular dysfunction seen in HFpEF.	Stimulate TGF-β and other profibrotic pathways in cardiac and vascular cells, contributing to interstitial fibrosis.	Act as DAMPs, activating innate immune pathways and sustaining chronic low-grade inflammation characteristic of HFpEF.	Mechanistic, preclinical (animal), observational human, and clinical studies
Lipoprotein-associated bioactive lipids (eicosanoids, oxylipins)	Derived from arachidonic and other polyunsaturated fatty acids; carried on lipoproteins; rapidly turned over.	Imbalanced vasodilator vs. vasoconstrictor eicosanoids modulate endothelial tone and reactivity in HFpEF.	Certain oxylipins promote fibroblast activation and extracellular matrix deposition.	Many species act as potent inflammatory mediators, linking dyslipidemia to systemic/myocardial inflammation in HFpEF.	Mechanistic, animal, observational human, and clinical studies

CE, cholesterol esters; CETP, cholesteryl ester transfer protein; DAMPs, damage-associated molecular patterns; eNOS, endothelial nitric oxide synthase; FFA, free fatty acids; HDL, high-density lipoprotein; HFpEF, heart failure with preserved ejection fraction; LCAT, lecithin-cholesterol acyltransferase; LDL, low-density lipoprotein; LPC, lysophosphatidylcholine; NO, nitric oxide; oxLDL, oxidized low-density lipoprotein; PC, phosphatidyl choline; PE, phosphatidyl ethanolamine; TAGs, triacylglycerols; TGF-β, transforming growth factor-beta; TLR, toll-like receptor; TyG, triglyceride-glucose.

## 5 Impact of dyslipidemia on endothelial dysfunction in HFpEF

The impact of dyslipidemia on endothelial dysfunction in HFpEF is multifaceted and involves several interconnected mechanisms. A common comorbidity in HFpEF patients, dyslipidemia is associated with microvascular impairment primarily through the action of saturated fatty acids (SFAs). Elevated plasma levels of SFAs induce endothelial dysfunction via toll-like receptor 4-dependent activation of NF-κB; this process significantly decreases NO availability, which is a crucial factor in maintaining vascular tone and endothelial health (54). Furthermore, SFAs activate the NLRP3 inflammasome within endothelial cells, resulting in the expression of IL-1β and an exacerbated inflammatory response (55). The subsequent accumulation of lipids in the arterial wall leads to the formation of atherosclerotic plaques; these not only physically obstruct blood flow but also serve as foci for ongoing inflammation and oxidative stress, further damaging the endothelium (56).

**Figure 2:**
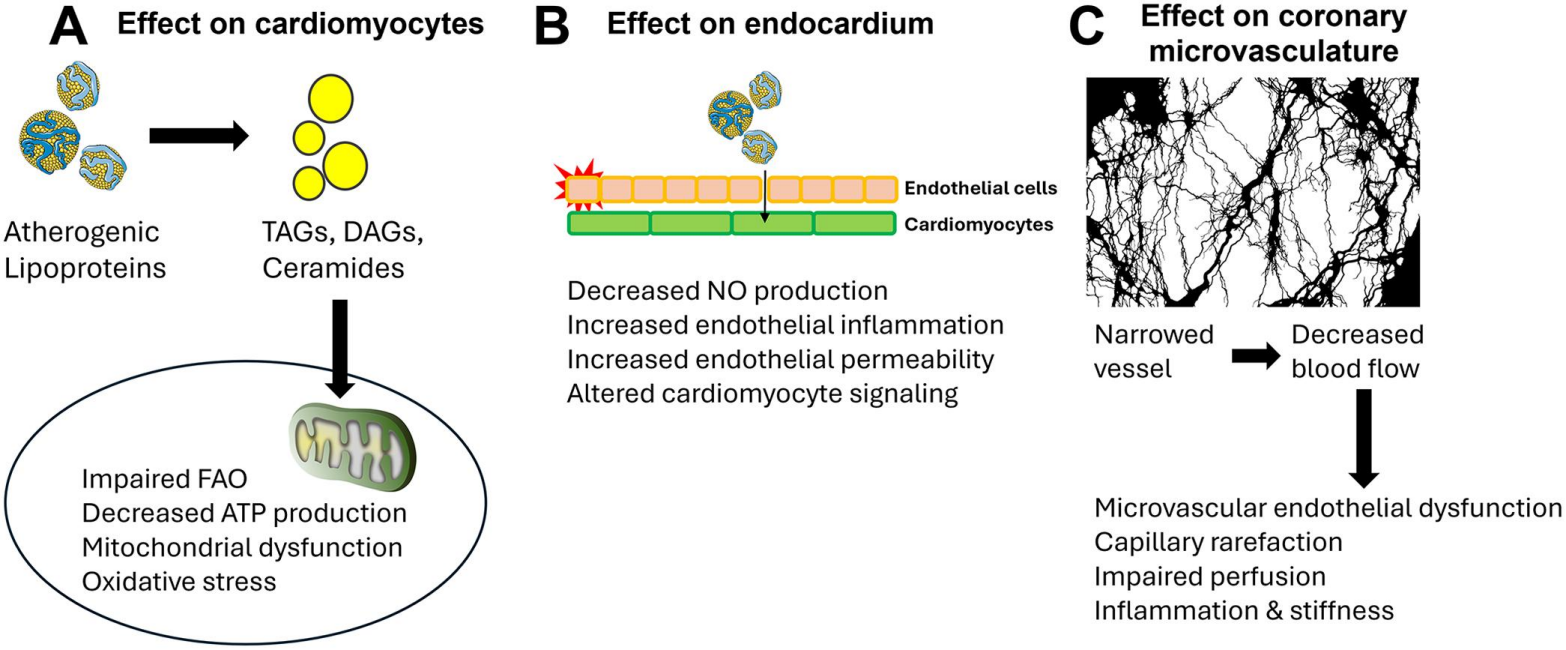
Proposed mechanism of atherogenic lipoprotein toxicity in HFpEF. This hypothetical model illustrates how highly modified atherogenic lipoproteins (such as electronegative VLDL) may contribute to HFpEF through coordinated effects as extrapolated from other cardiovascular disease models. **(A)** The proposed hypothesis is that atherogenic lipoproteins may directly impair cardiomyocyte metabolism and survival. The subsequent build-up of toxic lipid metabolites, such as triacylglycerols (TAGs), diacylglycerols (DAGs), and ceramides, impairs ATP production and energy metabolism. **(B)** In the proposed mechanism, endocardial dysfunction caused by atherogenic lipoproteins may alter cardiomyocyte signaling, oxygen delivery, and metabolic regulation. **(C)** With these cellular changes, blood flow may decrease in the capillary network surrounding the myocardium, thus impairing myocardial perfusion.

Other metabolic disturbances often seen in HFpEF patients, such as insulin resistance and obesity, can compound this endothelial impairment. These conditions contribute to a state of metabolic inflexibility in which the heart becomes overly reliant on carbohydrates for ATP production (57). This metabolic shift, combined with the low oxygen availability resulting from microvascular dysfunction, contributes to the cardiac energetic dysfunction characteristic of the disease. Notably, the resulting damage is not confined to the coronary vasculature but affects the systemic circulation, including the pulmonary vasculature, potentially contributing to the development of pulmonary hypertension, a common complication in HFpEF (19) (Figure 2). Ultimately, by promoting inflammation and oxidative stress, dyslipidemia seems to be strongly associated with microvascular dysfunction that is a hallmark of HFpEF pathophysiology. Understanding these specific mechanisms is crucial for developing targeted therapies for HFpEF (54, 58).

## 6 Effects of derangements in lipid metabolism on cardiac fibrosis in HFpEF

**Figure 3:**
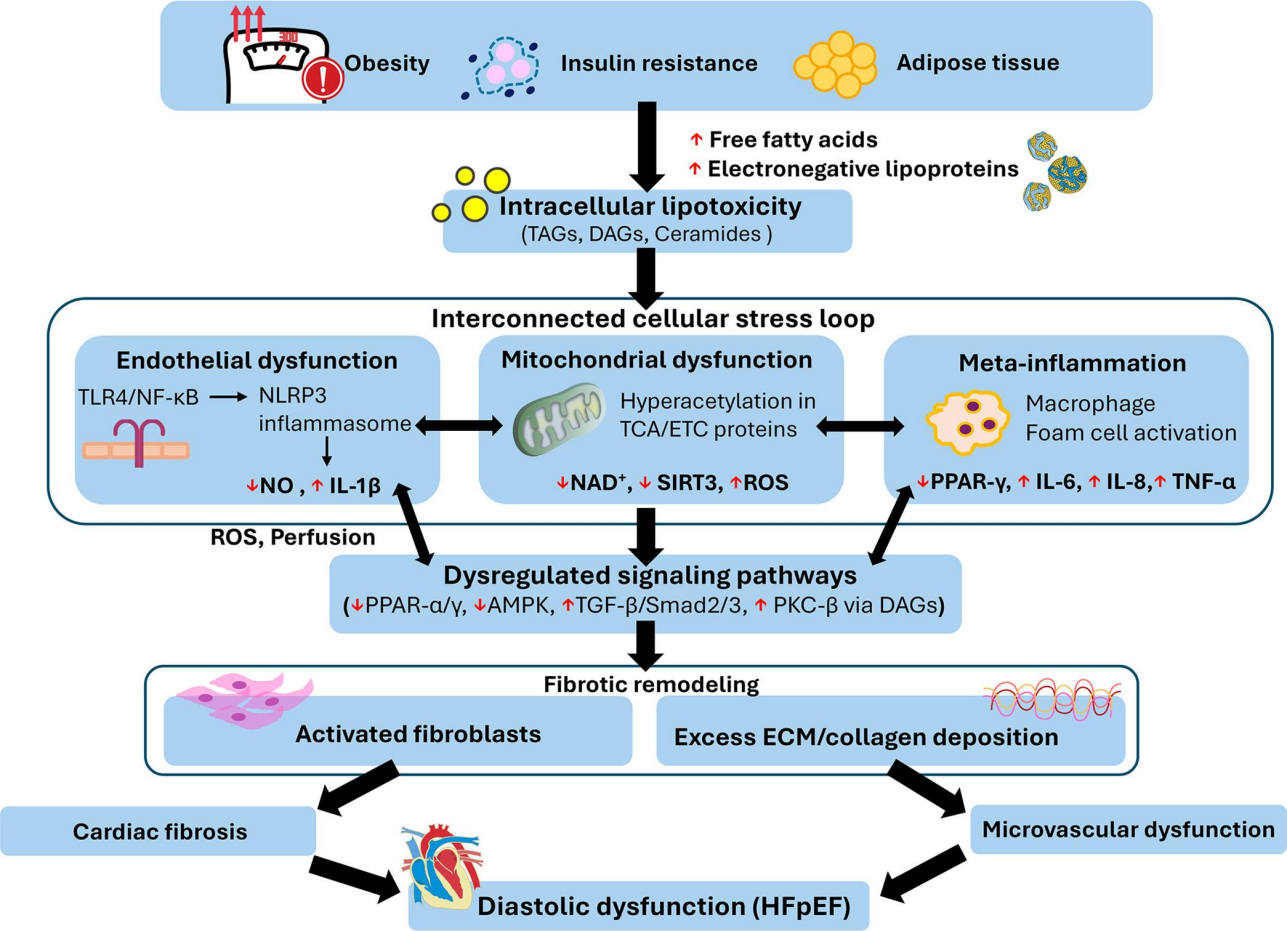
Established pathophysiology of general lipid-induced myocardial remodeling in HFpEF. Systemic metabolic stress and dyslipidemia promote the myocardial accumulation of saturated fatty acids and toxic lipid intermediates, including ceramides and diacylglycerols (DAGs). This lipotoxicity triggers an interconnected, recognized cascade of endothelial dysfunction, mitochondrial impairment, and meta-inflammation. In the endothelium, saturated fatty acids activate TLR4/NF-κB and NLRP3 inflammasomes, reducing nitric oxide (NO) bioavailability and promoting microvascular dysfunction. Concurrently, mitochondrial NAD^+^ depletion inactivates SIRT3, leading to protein hyperacetylation, impaired fatty acid oxidation, and excessive reactive oxygen species (ROS) generation. These metabolic and oxidative stressors drive immune cell recruitment and pro-inflammatory cytokine release [interleukin (IL)-1β, IL-6, and tumor necrosis factor (TNF)-α]. This inflammatory and lipotoxic milieu disrupts critical cellular signaling pathways, characterized by AMPK and PPAR downregulation alongside TGF-β/Smad2/3 and PKC-β activation, which collectively promote fibroblast-to-myofibroblast trans-differentiation. The resulting excessive extracellular matrix (ECM) deposition and collagen accumulation drive cardiac fibrosis, ultimately culminating in myocardial stiffness and the diastolic dysfunction characteristic of HFpEF.

In HFpEF hearts, a shift in substrate utilization results in an increased reliance on fatty acid uptake and glucose oxidation (59). This altered substrate utilization may indirectly promote fibrosis by exacerbating oxidative stress and mitochondrial dysfunction (37). Dysregulation of FAO and accumulation of lipid intermediates such as ceramides and DAGs can activate pro-fibrotic signaling pathways, including transforming growth factor-β (TGF-β) and connective tissue growth factor, ultimately leading to ECM deposition and fibrosis (60). This metabolic inflexibility reduces energetic efficiency and thereby contributes to the progression of cardiac dysfunction (Figure 3).

These metabolic alterations participate in a complex pathophysiological cascade that culminates in the development of cardiac fibrosis. Excess lipid deposition in cardiomyocytes can result in lipotoxicity, triggering various detrimental effects on cardiac function, including the promotion of fibrotic remodeling (61). Lipotoxicity-induced mitochondrial dysfunction in cardiomyocytes can contribute to increased production of ROS, which activate fibroblasts and increase the fibrotic burden in HFpEF (62).

Disrupted lipid metabolism in HFpEF is closely associated with a state of chronic low-grade inflammation, characterized by the recruitment of immune cells to the myocardium (57). This inflammatory milieu promotes fibroblast activation and excessive deposition of ECM proteins, further contributing to cardiac fibrosis. Pro-inflammatory macrophages, often referred to as “foam cells,” can release a variety of cytokines and chemokines that exacerbate fibroblast activation and collagen synthesis within the myocardium (61). The interplay between lipid-induced inflammation and myocardial fibrosis underscores the importance of targeting lipid metabolism as a therapeutic strategy to mitigate cardiac fibrosis and improve outcomes in HFpEF (63).

## 7 Cellular signaling pathways altered by abnormal lipids in HFpEF

In HFpEF, abnormal lipid accumulation and altered lipid metabolism are increasingly recognized as critical contributors to disease pathogenesis. Several key signaling pathways are implicated in the process, notably the TGF-β, PPAR, PKC, and AMP-activated protein kinase (AMPK) pathways, each playing distinct roles in cardiac remodeling and fibrosis (64) (Figure 3).

Elevated levels of lipotoxic intermediates activate TGF-β signaling, promoting fibroblast activation and ECM deposition (65). This, in turn, leads to increased myocardial fibrosis and contributes to diastolic dysfunction and increased myocardial stiffness in HFpEF patients (66). TGF-β activation further induces downstream signaling involving Smad2/3 proteins, which translocate to the nucleus and regulate the transcription of pro-fibrotic genes, including collagen and fibronectin, exacerbating fibrosis (67).

PPARs, particularly PPAR-α and PPAR-γ, are nuclear receptors that regulate lipid metabolism and inflammation. Abnormal lipid profiles in HFpEF patients are often associated with decreased PPAR-α expression, resulting in impaired FAO and increased lipid storage in cardiomyocytes (63). This downregulation of PPAR-α is accompanied by increased oxidative stress and inflammation, contributing to myocardial fibrosis and dysfunction. Similarly, decreased PPAR-γ activity has been linked to macrophage infiltration and pro-inflammatory cytokine production, further enhancing fibrotic processes (68).

The PKC signaling pathway is another important regulator of lipid-induced cardiac dysfunction. Accumulation of DAGs, a byproduct of altered lipid metabolism, can activate various isoforms of PKC, leading to a cascade of events that modulate ion channel function, contractility, and fibrosis (69). Activation of PKC-β, in particular, has been shown to enhance myocardial fibrosis by increasing the expression of matrix metalloproteinases and inhibiting collagen degradation (70). These changes contribute to maladaptive myocardial remodeling, which is characteristic of HFpEF.

AMPK, a central energy sensor in cardiomyocytes, is also affected by lipid abnormalities in HFpEF. AMPK activation is typically associated with improved FAO and reduced lipid accumulation; however, in HFpEF, increased lipotoxicity and mitochondrial dysfunction can contribute to impaired AMPK signaling (71). This impaired signaling results in decreased autophagy and increased oxidative stress, promoting fibrosis and myocardial dysfunction. The disrupted AMPK pathway in HFpEF highlights the intersection between energy metabolism and fibrotic remodeling.

Therefore, lipid abnormalities can disrupt cellular signaling pathways, leading to adverse structural and functional changes in the myocardium, ultimately contributing to the progression of HFpEF.

## 8 Inflammatory responses to abnormal lipids in HFpEF

Inflammatory responses to abnormal lipids play a crucial role in the pathophysiology of HFpEF and involve complex interactions between metabolic disturbances and immune system activation (Figure 3). These processes contribute to lipotoxicity, microvascular dysfunction, and energetic impairment in the heart, leading to HFpEF progression through various mechanisms. Abnormal lipid metabolism and accumulation trigger a chronic low-grade inflammatory state in HFpEF, termed metabolic inflammation or meta-inflammation (72).

Metabolic inflammation contributes significantly to HFpEF pathophysiology. Obesity and metabolic stress induce a chronic low-grade inflammatory state, characterized by the expansion of adipose tissue and increased release of chemokines that initiate immune cell recruitment (46). In obesity, metabolic alterations produce intermediates that activate immune responses, intensifying inflammatory activity (73). Disruptions in the tricarboxylic acid cycle associated with obesity, for example, can contribute to the accumulation of intermediates that drive pro-inflammatory macrophage polarization and activate inflammatory pathways (74).

**Figure 4:**
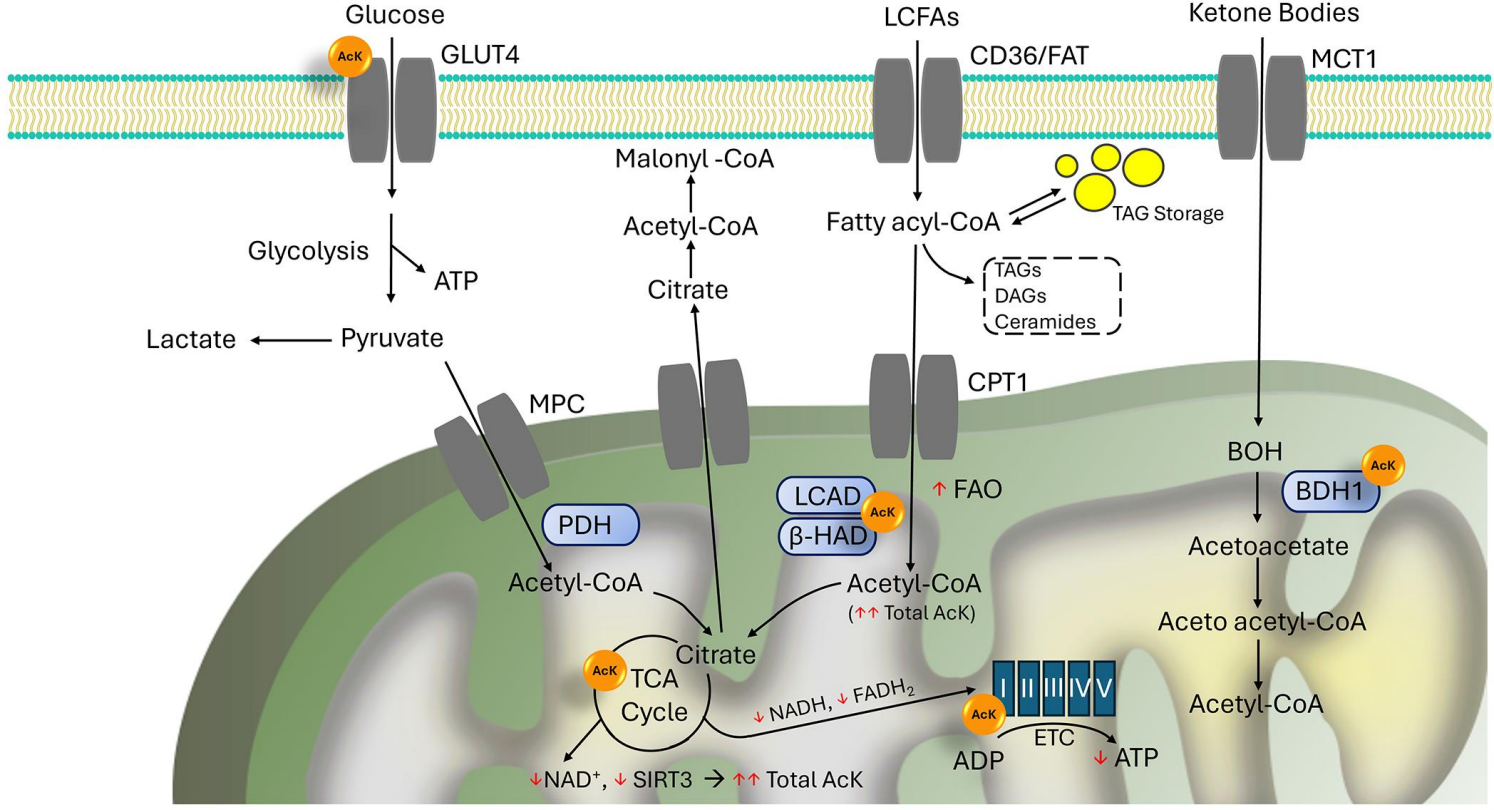
Mitochondrial hyperacetylation cycle as an established mechanism of metabolic failure in HFpEF. A comorbidity-driven reduction in myocardial NAD^+^ levels leads to widespread inactivation of NAD^+^-dependent deacetylase sirtuin 3 (SIRT3). The loss of SIRT3 activity prevents the removal of pathological lysine acetylation (AcK) tags from multiple mitochondrial proteins (indicated on enzymes in the TCA cycle and electron transport chain). This persistent “AcK” clog inhibits metabolic flux through these pathways, creating an energy shortage (ATP deficiency). This inhibition further depletes NAD^+^, perpetuating the hyperacetylation state and redirecting cellular lipids into the formation of toxic intermediates (DAGs/ceramides) that exacerbate cardiomyocyte dysfunction. BDH1: beta-hydroxybutyrate dehydrogenase 1; β-HAD: beta-hydroxyacyl-CoA dehydrogenase; CPT1: carnitine palmitoyltransferase 1; ETC: electron transport chain; FAO: fatty acid oxidation; LCAD: long-chain acyl-CoA dehydrogenase; LCFAs: long-chain fatty acids; MPC: mitochondrial pyruvate carrier; PDH: pyruvate dehydrogenase.

Excessive intramyocardial lipid accumulation leads to lipotoxicity. Mitochondrial dysfunction in HFpEF appears to be driven by a pathological accumulation of acetyl groups on mitochondrial proteins, a state of hyperacetylation (Figure 4). One proposed model suggests that comorbidity-driven reductions in the cofactor NAD^+^ can contribute to inactivation of the protective mitochondrial deacetylase SIRT3. This allows toxic lysine acetylation marks to physically accumulate on key metabolic enzymes (such as those in the tricarboxylic acid cycle and the electron transport chain), effectively clogging mitochondrial flux. This metabolic blockage further depletes NAD^+^ (perpetuating the cycle) and redirects lipids away from oxidation toward the formation of toxic intermediates like DAGs and ceramides, which directly compromise cardiomyocyte survival and function (Figure 4). The accumulation of toxic lipid intermediates activates inflammatory signaling pathways including the toll-like receptor 4 pathway and induces pro-inflammatory cytokine production, such as interleukin-6, TNF-α, and interleukin-1β (75). These inflammatory mediators contribute to cardiac dysfunction and remodeling in HFpEF. Epicardial adipose tissue releases pro-inflammatory adipokines and cytokines, which promote local inflammation and oxidative stress in the myocardium (76). While clinical evidence supports this model, the exact mechanisms of epicardial adipose tissue-induced myocardial dysfunction in HFpEF are not clear.

Collectively, these processes result in microvascular dysfunction as seen in HFpEF pathogenesis (57). The interplay between metabolic disturbances and inflammatory burden is complex and central to HFpEF pathophysiology. Metabolic stress induces a systemic pro-inflammatory state, in which lipids act as inflammatory molecules and participate in the recruitment of immune cells in the HFpEF myocardium (77). Among abnormal lipids, electronegative lipoproteins have emerged as significant mediators because of their pro-inflammatory and atherogenic properties.

## 9 Types and *in vivo* production of electronegative lipoproteins

Understanding the interplay between lipids and inflammation requires studying lipoproteins, the main carrier of lipids. In recent decades, lipoproteins have been not only quantified but also qualified based on their physicochemical modifications. For example, L5, sometimes called LDL(-), is a heterogeneous group of circulating lipoproteins characterized by their increased negative charge compared to native LDL; they have been implicated in various atherogenic processes (78). The *in vivo* production of electronegative lipoproteins involves several mechanisms that can result in different subtypes.

One of the primary types of electronegative LDL is oxidized LDL, which is formed through lipid peroxidation and apolipoprotein B-100 (apoB-100) alteration (79). This oxidative modification can occur in the subendothelial space of the arterial wall or in the circulation, particularly under conditions of increased oxidative stress. Another type of electronegative LDL is glycated LDL, which is produced through non-enzymatic glycation of lysine residues on apoB-100. This process is especially prevalent in diabetic patients because of their hyperglycemia (80). LDL(-) can also be generated through enzymatic modifications, such as hydrolysis of phospholipids at the sn-2 position to generate lysophospholipids and free fatty acids, which contribute to the increased negative charge (81). Bancells et al. identified up to 28 different proteins in LDL fractions, with LDL(-) showing a higher content of most minor proteins compared to native LDL. The increased presence of apolipoproteins such as apoE, apoA-I, apoC-III, apoA-II, apoD, apoF, and apoJ in LDL(-) could be related to its physicochemical characteristics and atherogenic properties (79). Chen et al. developed a different method to separate LDL into five subfractions (L1-L5) based on electronegativity (82–84). The most electronegative fraction, L5, is highly O-glycosylated on apoB-100 and apoE, and terminal sialic acid residues contribute to its increased electronegativity and hydrophilicity (85).

*In vivo* production of electronegative lipoproteins can be increased in various pathological conditions. For example, patients with metabolic disorders such as diabetes, obesity, and metabolic syndrome tend to have higher levels. Chronic kidney disease, familial hypercholesterolemia, and systemic lupus erythematosus are also associated with increased production of electronegative LDL (79, 80). The heterogeneity of these particles and their complex formation processes highlight the need for further research to fully elucidate their role in atherogenesis and systemic inflammation. Understanding the types and mechanisms of *in vivo* production of electronegative lipoproteins is crucial for developing targeted therapies and biomarkers for cardiovascular diseases.

## 10 Hypothesis and future directions: the potential role of electronegative VLDL (V5) in HFpEF

Although the detrimental effects of general lipid abnormalities in HFpEF are well-documented, the specific impact of VLDL subfractions remains an emerging field of study. Currently, direct clinical evidence evaluating V5 specifically in HFpEF cohorts is limited. However, based on extensive research demonstrating the severe lipotoxic and pro-inflammatory properties of V5 in related metabolic syndromes (86–93), we hypothesize that V5 may play a highly localized, disproportionate role in driving the microvascular inflammation and myocardial fibrosis characteristic of HFpEF.

In HFpEF, the myocardial metabolism changes from primarily FAO to glycolysis. Because VLDL is the main TAG transporter, investigating the quality of VLDL particles is crucial. Similar to LDL, V5 is the most electronegative VLDL fraction. Specific studies on V5 in HFpEF are limited, but insights can be drawn from related research on electronegative lipoproteins and HFpEF pathophysiology.

HFpEF is associated with metabolic disorders including dyslipidemia, and the presence of abnormal lipoproteins, such as V5, may contribute to its pathophysiology through various mechanisms. Electronegative lipoproteins, including V5, induce inflammatory responses (86, 94, 95). Chen et al. reported that the combined electronegativity of L5 and V5 (L5 + V5) correlated with coronary heart disease risk in asymptomatic individuals (88). This suggests that V5, along with L5, may contribute to the chronic low-grade inflammation observed in HFpEF. Furthermore, V5 may promote endothelial dysfunction, a key feature of HFpEF. In cultured human aortic endothelial cells, Chen et al. showed that treatment with L5 + V5 induced significantly more senescence-associated β-Gal activity than treatment with less electronegative fractions (88). This endothelial senescence may contribute to the microvascular dysfunction observed in HFpEF. Electronegative lipoproteins also induce oxidative stress, which plays a crucial role in cardiac remodeling and dysfunction in HFpEF (16). V5 may contribute to this process by promoting the production of ROS in various cell types, including cardiomyocytes and endothelial cells (93).

Lipotoxicity, another critical aspect of HFpEF pathophysiology, is characterized by excessive accumulation of lipids in cardiomyocytes (16). As a modified form of VLDL, V5 may contribute to lipid accumulation and subsequent metabolic stress in cardiac tissue. In a study by Chen et al., preliminary data showed that V5 was rich in apoCIII, signaling a possible mechanism for TAGs accumulation in cells (93). However, direct evidence remains limited for V5's role in HFpEF. Future research should focus on investigating the direct effects of V5 on cardiomyocyte function, particularly diastolic dysfunction. Additionally, quantifying V5 levels in HFpEF patients and their possible relationship in HFpEF severity or prognosis could provide valuable insights. Although the specific importance of V5 in HFpEF is not fully elucidated, existing research on electronegative lipoproteins and HFpEF pathophysiology suggests that V5 may play a significant role and can contribute to novel therapeutic strategies (87).

## 11 Lipidomic profiling and clinical phenotyping in HFpEF

### 11.1. Shift to advanced lipidomics

Historically, the evaluation of lipid abnormalities in heart failure has relied on standard lipid panels measuring total cholesterol, LDL, high-density lipoproteins, and TAGs. However, these conventional metrics often fail to capture the complex metabolic derangements driving HFpEF pathophysiology. Recent advances in mass spectrometry-based lipidomics have revolutionized this landscape, enabling the simultaneous quantification of thousands of individual lipid species (96). This high-throughput profiling provides a highly granular view of the cardiac and systemic lipidome, revealing that HFpEF is characterized by specific lipidomic signatures that standard clinical panels overlook (3).

### 11.2. Distinct lipid signatures in HFpEF

Lipidomic studies have consistently identified specific lipid classes associated with the severity of diastolic dysfunction and adverse outcomes in HFpEF (97). Elevated circulating and myocardial levels of sphingolipids, particularly ceramides, have emerged as potent biomarkers. Ceramides are known to induce lipotoxicity, promote mitochondrial dysfunction, and exacerbate myocardial stiffness, directly correlating with adverse echocardiographic parameters (98). Additionally, alterations in DAGs and specific species of phosphatidylcholines have been linked to the pro-inflammatory and pro-fibrotic signaling cascades detailed previously (3). By mapping these specific lipid species, researchers can better quantify the degree of metabolic stress and structural remodeling occurring at the cellular level.

### 11.3. Clinical applications: lipidomics-guided phenotyping

Beyond serving as diagnostic biomarkers, lipidomic profiling has profound potential for clinical phenotyping. HFpEF is a highly heterogeneous syndrome with varying underlying etiologies. Lipidomics allows for the stratification of patients into distinct metabolic phenotypes, such as the “obese-metabolic” HFpEF phenotype, which is characterized by a high burden of lipotoxicity and meta-inflammation, versus non-metabolic variants (99). This lipidomics-guided phenotyping is a critical step toward precision medicine. By identifying the specific lipid derangements driving an individual patient's disease, clinicians may soon be able to tailor therapeutic interventions, such as targeted metabolic modulators, sodium-glucose co-transporter 2 (SGLT2) inhibitors, or specific anti-inflammatory agents, to yield the highest clinical benefit (100). Ultimately, integrating lipidomic profiling into routine clinical research offers a powerful tool for unraveling HFpEF heterogeneity and optimizing patient-specific treatment strategies.

## 12 Therapeutic strategies targeting lipid abnormalities in HFpEF

Therapeutic strategies targeting lipid abnormalities in HFpEF are an emerging area of research, given the growing evidence that altered lipid metabolism contributes to disease progression. These strategies focus on correcting lipid dysregulation, reducing lipotoxicity, and improving myocardial energy efficiency to alleviate cardiac fibrosis and dysfunction (101). Proposed approaches involve the use of pharmacological agents, lifestyle interventions, and experimental therapies aimed at modulating key pathways involved in lipid metabolism, including the PPAR, AMPK, and TGF-β pathways (Table 2).

**Table 2:** Cellularly targeted, lipid-focused therapeutic strategies in HFpEF and their links to lipotoxicity and cell injury.

Modality/class	Example agents/interventions	Primary cellular/lipid-related targets	Clinical outcome in HFpEF	Mechanistic relevance to abnormal lipids and cellular dysfunction
SGLT2 inhibitors	Empagliflozin, dapagliflozin	Reduce epicardial adipose tissue inflammation, modulate NHE, and improve myocardial energetics.	Lower HF hospitalizations and improve clinical status across preserved EF spectrum.	Limit lipotoxicity, improve mitochondrial efficiency, dampen inflammatory signaling, and improve ventricular-arterial coupling.
ARNI	Sacubitril/valsartan	Improves NP-cGMP/PKG signaling and endothelial function; limits hypertrophy/fibrosis.	Improves symptoms and lowers hospitalizations in lower-EF HFpEF.	cGMP-PKG activation reduces cardiomyocyte stiffness and may counter ceramide/ROS-driven titin hypophosphorylation and fibrosis.
MRAs	Spironolactone, eplerenone, finerenone	Reduce myocardial and vascular fibrosis/inflammation; modulate ECM and macrophage phenotype.	Reduce HF hospitalization risk in selected HFpEF subsets.	Attenuate aldosterone-driven fibroblast activation and collagen deposition, partially offsetting downstream consequences of lipotoxic and ceramide signaling.
RAAS blockers (ARB/ACEi)	Candesartan, losartan, others	Reduce Ang II–mediated oxidative stress/inflammation, and fibrosis in microvasculature and myocardium.	Fair benefit on HF hospitalization; background therapy in many HFpEF phenotypes.	Indirectly reduce lipid-induced endothelial injury and fibrosis by dampening Ang II-ROS/NF-κB signaling.
Diuretics	Loop and thiazide(-like) diuretics	Relieve congestion and wall stress; little direct effect on lipid biology.	Symptomatic improvement without proven mortality benefit.	Lower wall stress/microvascular congestion; indirectly save endothelium, no direct lipotoxic effect.
Weight loss and metabolic interventions	Biguanides, caloric restriction, structured weight loss, GLP-1 receptor agonists	Reduce adiposity, ectopic fat, and systemic lipotoxicity; improve insulin sensitivity.	Improve exercise capacity and quality of life in obese HFpEF.	Reduce FFA/TAGs flux to heart and vessels; lower ceramides, inflammation, and endothelial dysfunction.
Exercise training/ cardiac rehab	Aerobic and combined training programs	Improves skeletal muscle oxidative capacity, endothelial function, and inflammatory profile.	Increases peak VO₂ and functional capacity in HFpEF.	Enhances microvascular function, reduces inflammatory cytokines, and may partially reverse peripheral lipotoxic skeletal muscle phenotype.
Lipid-modifying therapies	Statins, fibrates, PCSK9 inhibitors, TAGs-lowering therapies	Lower LDL-C and atherogenic lipoproteins; modulate vascular inflammation and oxidative stress.	Outcome benefit established in ASCVD; HFpEF data limited but mechanistically supportive.	Reduce burden of oxidized LDL and pro-inflammatory lipids, improving endothelial and coronary microvascular function.
Targeted anti-ceramide/sphingolipid strategies (emerging)	Experimental ceramide synthesis inhibitors, sphingolipid modulators	Reduce ceramide accumulation in cardiomyocytes/endothelium; modulate cell death and fibrosis.	Better vascular function and reduced fibrosis in preclinical data; outcomes awaited.	Aim directly at a key lipotoxic axis implicated in endothelial dysfunction, myocardial stiffness, and fibrotic remodeling in HFpEF.
Anti-inflammatory/ antifibrotic agents (emerging)	TGF-β modulators, inflammasome inhibitors, antifibrotics	Attenuate macrophage activation, cytokine release, fibroblast activation, and ECM deposition.	Under investigation; may be most relevant in inflammatory-lipotoxic HFpEF phenotypes.	Target effects of abnormal lipids (oxidized phospholipids, ceramides) that drive immune activation and fibrogenesis.
Myosin/sarcomere modulators (emerging)	Mavacamten, other myosin inhibitors	Improve active relaxation and diastolic properties at the myocyte level.	Biomarker and symptom improvement (early data); outcome data pending.	Target sarcomere dysfunction and diastolic abnormalities; secondary to lipotoxic/fibrotic remodeling, not lipid biology.

ACEi, angiotensin-converting enzyme inhibitors; ASCVD, atherosclerotic cardiovascular disease; Ang II, angiotensin II; ARB, angiotensin receptor blocker; ARNI, angiotensin receptor/neprilysin inhibitor; ASCVD, atherosclerotic cardiovascular disease; ECM, extracellular matrix; EF, ejection fraction; FFA, free fatty acid; GLP-1, glucagon-like peptide-1; HF, heart failure; HFpEF, heart failure with preserved ejection fraction; LDL, low-density lipoprotein; MRAs, mineralocorticoid receptor antagonists; NHE, sodium-hydrogen exchange, leucine-rich repeat-containing, pyrin domain-containing 3; NP-cGMP/PKG, natriuretic peptide-cyclic GMP/protein kinase G; PCSK9, proprotein convertase subtilisin-kexin type 9; RAAS, renin-angiotensin-aldosterone system; ROS, reactive oxygen species; SGLT2, sodium-glucose co-transporter 2; TAGs, triacylglycerols; TGF-β, transforming growth factor-beta; VO_2_, oxygen consumption.

### 12.1 Pharmacological approaches

***PPAR agonists*:** PPARs are nuclear receptors that regulate fatty acid metabolism and glucose homeostasis. Agonists targeting PPAR-α and PPAR-γ, such as fibrates and thiazolidinediones, have shown promise in modulating lipid metabolism and reducing lipotoxicity in HFpEF. PPAR-α agonists like fenofibrate enhance FAO and decrease TAGs levels, potentially mitigating lipotoxic damage to cardiomyocytes (102). Meanwhile, PPAR-γ agonists like pioglitazone improve insulin sensitivity and reduce inflammation, which are critical for managing metabolic syndrome-associated HFpEF (103). However, clinical trials assessing the benefits of these agents in HFpEF populations have shown mixed results, indicating the need for further research to optimize their use in this setting (104–107).

***AMPK activators*:** AMPK is a crucial energy sensor in cells, and its activation promotes FAO, mitochondrial biogenesis, and autophagy, thereby maintaining cardiac energy balance. Drugs such as metformin, an AMPK activator, have been studied for their potential benefits in HFpEF due to their ability to enhance lipid metabolism and reduce myocardial fibrosis (108). Moreover, recent preclinical studies using direct AMPK activators, such as 5-aminoimidazole-4-carboxamide ribonucleotide, have demonstrated reduced lipid accumulation and improved diastolic function in animal models of HFpEF (109).

***SGLT2 inhibitors*:** SGLT2 inhibitors, originally developed for diabetes management, have shown unexpected cardioprotective effects in heart failure. While agents such as empagliflozin and dapagliflozin lower glucose and lipid levels, recent landmark clinical trials, specifically EMPEROR-Preserved and DELIVER, have firmly established them as a foundational therapy by demonstrating significant reductions in hospitalization rates and cardiovascular events for HFpEF patients (110, 111). Beyond glycemic control, the cardiometabolic benefits of SGLT2 inhibitors are highly pleiotropic and do not rely on a single pathway. Metabolically, they promote a shift toward ketogenesis, which provides an efficient alternative energy substrate for the failing heart and helps reduce myocardial lipotoxicity. However, other profound physiological mechanisms are equally critical to their efficacy in HFpEF. These include the direct modulation of the myocardial sodium-hydrogen exchanger, which mitigates deleterious intracellular sodium and calcium overload, thereby improving cardiomyocyte relaxation. Additionally, SGLT2 inhibitors are known to exert potent anti-inflammatory effects, notably by reducing the volume and inflammatory signaling of epicardial adipose tissue, a key driver of microvascular dysfunction in HFpEF. Hemodynamically, they also reduce cardiac afterload and improve ventricular-arterial coupling. Together, these multifaceted metabolic, structural, and hemodynamic mechanisms act synergistically to ameliorate the core deficits of HFpEF.

***GLP-1 receptor agonists*:** Glucagon-like peptide-1 (GLP-1) receptor agonists have recently emerged as a highly promising therapeutic avenue, fundamentally shifting the treatment paradigm for the obese/cardiometabolic phenotype of HFpEF. Historically, obesity was viewed merely as a comorbidity; however, contemporary evidence now positions profound obesity and visceral adiposity potentially as central drivers of systemic inflammation, lipotoxicity, and mechanical myocardial restriction. Consequently, intentional weight loss via targeted pharmacological obesity treatment is now a potent, disease-modifying strategy as definitively demonstrated in the recent landmark STEP-HFpEF trial, which investigated the use of semaglutide in obese patients with HFpEF (112). Treatment with semaglutide substantially reduced body weight, dramatically improved heart failure-related symptoms, and significantly enhanced exercise capacity (measured via the 6-minute walk distance) compared to placebo.

The cardioprotective mechanisms of GLP-1 receptor agonists extend beyond simple mechanical unloading due to weight loss. At the cellular level, these agents exert profound systemic and localized anti-inflammatory effects. By significantly reducing visceral and epicardial adipose tissue burden, semaglutide directly diminishes the paracrine release of pro-inflammatory adipokines and toxic lipid intermediates that drive coronary microvascular endothelial dysfunction and myocardial fibrosis (113–115). Furthermore, GLP-1 receptor agonists improve systemic insulin sensitivity and glucose metabolism, thereby helping to correct the metabolic mismatch and systemic lipid oversupply that fuel intramyocardial lipotoxicity. Together, these effects underscore the critical importance of targeting the root adiposity-driven inflammatory pathways to modify disease progression in cardiometabolic HFpEF.

***Statins*:** Statins are foundational for preventing cardiovascular events by reducing LDL cholesterol and improving endothelial function. However, multiple large randomized clinical trials and comprehensive meta-analyses have consistently failed to demonstrate a reduction in heart failure events or mortality with statin therapy in HFpEF patients. This divergence in efficacy highlights the fundamental pathophysiological differences between heart failure phenotypes. In HFrEF, which is frequently driven by ischemic heart disease and macrovascular coronary atherosclerosis, statins confer a protective effect by stabilizing plaques, reducing ischemic events, and preventing further adverse myocardial remodeling (116, 117). In contrast, HFpEF is primarily driven by systemic metabolic dysfunction, coronary microvascular inflammation, and myocardial steatosis rather than obstructive epicardial coronary disease. Because statins primarily target hepatic cholesterol synthesis and circulating LDL-C, they fail to adequately address the intramyocardial accumulation of toxic lipid species that directly cause diastolic dysfunction. Although statins have not shown clear clinical benefit in HFpEF, lipids still play a crucial role in its pathophysiology. However, in HFpEF, intracellular lipid intermediates (e.g., ceramides) appear to be more critical drivers of the disease than circulating LDL-C (118, 119). While some mechanistic studies proposed that statins might pleiotropically attenuate myocardial fibrosis (120), the clinical consensus indicates that statins do not provide targeted HFpEF benefits. Statins remain recommended in HFpEF populations solely for the management of comorbid coronary artery disease.

### 12.2 Lifestyle and dietary interventions

Diet and lifestyle modifications are foundational strategies for managing lipid abnormalities in HFpEF. Dietary approaches such as the Mediterranean diet, which is rich in monounsaturated and polyunsaturated fatty acids, have been associated with improved lipid profiles and reduced cardiovascular risk (121). Exercise training, including both aerobic and resistance modalities, can enhance FAO and improve insulin sensitivity, thereby reducing lipid accumulation and myocardial stiffness (122). Weight management is also crucial, as obesity is a common comorbidity in HFpEF. Studies suggest that intentional weight loss can improve diastolic function and reduce myocardial lipid content, potentially alleviating HFpEF symptoms (123). Combining dietary modifications with structured exercise programs may provide synergistic benefits by addressing both metabolic and mechanical contributors to HFpEF.

### 12.3 Experimental therapies and future directions

Emerging therapies targeting specific lipid-related pathways are currently under investigation. These include inhibitors of ceramide synthesis, which aim to reduce lipotoxicity and myocardial inflammation, and agents targeting the TGF-β pathway to attenuate fibrosis (124). Additionally, gene therapy approaches using adeno-associated viruses to deliver AMPK or PPAR activators directly to the heart are being explored as potential strategies to restore lipid homeostasis and improve cardiac function (125, 126). MicroRNAs involved in lipid metabolism and fibrosis regulation, such as miR-33 and miR-21, are also being evaluated as therapeutic targets in HFpEF. Modulating these microRNAs using antisense oligonucleotides or small molecule inhibitors could provide novel means to correct lipid abnormalities and mitigate fibrotic remodeling (125, 127, 128).

Overall, the therapeutic landscape for targeting lipid abnormalities in HFpEF is evolving. Given the heterogeneity of HFpEF, a personalized approach that considers the unique lipid metabolic profile and comorbidities of each patient may be essential for optimizing treatment outcomes.

## 13 Understanding the cellular complexity of HFpEF pathogenesis through lipid research

The heterogeneous nature of HFpEF has necessitated a deeper examination of its cellular underpinnings, particularly how lipid disturbances contribute to its pathogenesis. The insights of myocardial metabolism and lipid dysregulation presented earlier can be consolidated to show how aberrant lipid profiles alter cellular homeostasis. Dysregulated lipids, including electronegative lipoproteins and abnormal VLDL subtypes, initiate inflammatory cascades, endothelial dysfunction, and fibrosis, all of which are hallmarks of HFpEF. By exploring the nuanced crosstalk between lipids and cellular signaling pathways, we can better understand how lipid-driven processes exacerbate cellular dysfunction and create an environment for HFpEF development. This integrated perspective not only highlights the pathophysiological relevance of lipid abnormalities but also offers new directions for targeted therapeutic strategies aimed at restoring lipid homeostasis and mitigating cellular damage in HFpEF.

## 14 Current knowledge gaps and limitations

Although systemic lipid abnormalities are established contributors to HFpEF, the specific role of highly modified lipoprotein subfractions, particularly V5, remains hypothetical and requires direct validation. A major limitation of the current literature is that evidence demonstrating the pro-inflammatory, lipotoxic, and profibrotic effects of V5 is predominantly derived from models of atherosclerosis, macrovascular coronary artery disease, and isolated endothelial cell assays. Because HFpEF is primarily a microvascular, myocardial, and systemic metabolic syndrome, distinct from the macrovascular, plaque-driven pathology of classical coronary artery disease, extrapolating these findings directly to HFpEF pathogenesis requires caution.

Currently, direct evidence linking V5 to HFpEF is extremely limited. There is a lack of clinical studies measuring circulating V5 levels specifically within phenotype-defined HFpEF patient cohorts. Consequently, it is unknown whether V5 is disproportionately elevated in HFpEF compared to other metabolic syndromes, or if it correlates with the severity of diastolic dysfunction. Acknowledging these limitations is crucial. Distinguishing between established, general lipotoxic mechanisms in HFpEF and hypothesis-generating observations regarding V5 ensures that the current evidence base is accurately represented and interpreted.

## 15 Future research directions

As our understanding of the metabolic determinants of HFpEF evolves, future research must pivot from generalized observations of dyslipidemia toward highly targeted, phenotype-specific investigations to bridge existing knowledge gaps. Several key frontiers will define the next era of HFpEF lipid research as described below.

### 15.1 Advanced spatial lipidomics and biomarker discovery

Traditional plasma lipid panels provide an incomplete picture of myocardial lipotoxicity. Future studies should leverage advanced mass spectrometry-based lipidomics and spatial transcriptomics to map the precise localization of toxic lipid intermediates (e.g., ceramides, DAGs) within the human myocardium (39, 129, 130). Establishing specific circulating lipidomic signatures that reliably mirror this intracellular lipotoxicity will be crucial for developing non-invasive biomarkers that can identify high-risk patients before irreversible fibrotic remodeling occurs. Furthermore, large-scale, prospective observational studies are urgently needed to quantify specific modified lipoproteins, such as V5, in HFpEF patients to evaluate their potential utility as disease-specific biomarkers.

### 15.2 Precision medicine in cardiometabolic HFpEF

Given the heterogeneity of HFpEF, future clinical trials must prioritize phenotype-guided enrollment. Specifically, investigating the distinct metabolic responses of the obese/cardiometabolic HFpEF phenotype compared to age-related or hypertensive variants will be essential (10, 131). Research should focus on how distinct systemic lipid environments dictate these varying pathophysiological trajectories, moving the field closer to true precision medicine.

### 15.3 Targeted mechanistic and *in vivo* modeling

To definitively evaluate the lipotoxic hypotheses suggested by published studies, investigators must refine experimental models. Advanced animal models of HFpEF (such as double-hit metabolic/hypertensive models) must be developed to test whether the targeted infusion or endogenous accumulation of modified lipoproteins (such as V5) directly induces myocardial stiffness and microvascular inflammation. Furthermore, the focus of *in vitro* studies must shift from macrovascular endothelial cells to cardiac-specific microvascular endothelial cells and primary cardiomyocytes to accurately map localized cardiac effects.

### 15.4 Novel therapeutics and combinatorial strategies

Although agents such as SGLT2 inhibitors and GLP-1 receptor agonists have revolutionized the treatment of cardiometabolic HFpEF, their synergistic potential requires rigorous clinical evaluation. Recently published observational studies suggest that combination therapy reduces heart failure exacerbations, hospitalizations, and mortality compared to SGLT2 inhibitors alone. Future research should investigate whether combining these therapies offers compounded benefits in resolving metabolic mismatch and epicardial adipose tissue inflammation (132). Furthermore, there is a critical need to develop and test novel pharmacological agents that directly target intracellular lipid handling, such as specific inhibitors of ceramide biosynthesis pathways (e.g., myriocin, CERS6 inhibitors, fenretinide) or agents designed to neutralize highly modified, pro-inflammatory lipoproteins such as V5, L5, and other oxidized/modified lipoprotein subfractions (133–137). Ultimately, bridging the gap between molecular lipid research and clinical trial design will be paramount in discovering disease-modifying therapies capable of halting or reversing the progression of HFpEF.

## 16 Conclusion

This review delineates the complex role of lipid abnormalities in the pathogenesis of HFpEF, emphasizing the intricate interplay between lipid dysregulation and cellular dysfunction. Although HFpEF is traditionally associated with systemic comorbidities such as hypertension, obesity, and diabetes, emerging evidence highlights the importance of further exploring the cellular and metabolic disturbances that contribute to its progression.

Our analysis indicates that abnormal lipid metabolism and the intracellular accumulation of toxic lipid intermediates appear to act as a central driver for HFpEF pathology. Furthermore, we hypothesize that highly modified electronegative lipoproteins such as VLDL subfraction V5 may emerge as previously unrecognized contributors. These toxic lipid intermediates and modified lipoproteins can potentiate inflammatory responses, endothelial dysfunction, and myocardial fibrosis, as previously observed in both clinical and experimental cardiovascular studies (138). This process is mediated by altered lipid signaling pathways that disrupt the balance between pro- and anti-inflammatory signaling, thereby exacerbating the cardiac inflammatory milieu (80). Lipid-driven inflammatory processes, in turn, induce a state of endothelial activation and dysfunction, which compromises vascular reactivity and myocardial perfusion (17, 139). This cascade ultimately culminates in structural changes that impair myocardial relaxation and filling capacity, key characteristics of HFpEF. Additionally, dysregulated lipid metabolism may drive cardiac fibrosis through multiple mechanisms, including activation of profibrotic signaling pathways such as TGF-β (140). These pathways, once triggered, promote excessive ECM deposition, altering myocardial stiffness and compliance. Our findings on the cellular signaling pathways disrupted by abnormal lipids suggest that interventions targeting these pathways could potentially modulate fibrotic responses and improve myocardial function in HFpEF patients. Given the centrality of lipid abnormalities in HFpEF pathophysiology, therapeutic strategies targeting intracellular lipotoxicity and systemic metabolic dysfunction, rather than just correcting circulating dyslipidemia, should be prioritized. Although traditional lipid-lowering therapies such as statins have not demonstrated targeted clinical benefits in HFpEF, modern agents that broadly modulate systemic metabolism and reduce lipotoxicity, such as SGLT2 inhibitors, GLP-1 receptor agonists, and PPAR-γ agonists, have shown significant success in reducing the inflammatory burden (110). Furthermore, developing novel therapies targeting specific pathological lipid species, such as electronegative lipoproteins (e.g., V5) or modified LDL particles, could further improve outcomes for HFpEF patients (141–143). As our understanding of the molecular signatures and *in vivo* behavior of these lipids improves, precision medicine approaches integrating lipidomics could be valuable in identifying high-risk HFpEF phenotypes and personalizing treatment strategies.

This review consolidates proposed mechanisms and emerging evidence regarding the contributions of abnormal lipids to HFpEF at a cellular level, providing a framework for future research to explore targeted therapies that address lipid dysregulation. Bridging the gap between lipid metabolism and cellular dysfunction may be pivotal in better understanding the complex pathogenesis of HFpEF and identifying potential therapeutic options for this challenging disease.

## References

[1] Luchi RJ Snow E Luchi JM Nelson CL Pircher FJ. Left ventricular function in hospitalized geriatric patients. J Am Geriatr Soc. (1982) 30(11):700–5. 10.1111/j.1532-5415.1982.tb01983.x 7130576

[2] McDonagh TA Metra M Adamo M Gardner RS Baumbach A Böhm M. 2021 ESC guidelines for the diagnosis and treatment of acute and chronic heart failure. Eur Heart J. (2021) 42(36):3599–726. 10.1093/eurheartj/ehab368 34447992

[3] Jovanovic N Foryst-Ludwig A Klose C Da Conceicao CR Alasfar L Birkner T. An altered plasma lipidome-phenome network characterizes heart failure with preserved ejection fraction. ESC Heart Fail. (2024) 11(3):1553–66. 10.1002/ehf2.14654 38243357 PMC11098625

[4] Cotter G Petrie MC Butler J Davison B Chioncel O Biegus J. Obesity and inflammation in chronic and acute heart failure. Heart Fail Rev. (2025) 30(5):923–30. 10.1007/s10741-025-10518-x 40319221

[5] Aboumsallem JP Muthuramu I Mishra M De Geest B. Cholesterol-Lowering gene therapy prevents heart failure with preserved ejection fraction in obese type 2 diabetic mice. Int J Mol Sci. (2019) 20(9):2222. 10.3390/ijms20092222 31064116 PMC6539537

[6] Daneii P Neshat S Mirnasiry MS Moghimi Z Dehghan Niri F Farid A. Lipids and diastolic dysfunction: recent evidence and findings. Nutr Metab Cardiovasc Dis. (2022) 32(6):1343–52. 10.1016/j.numecd.2022.03.003 35428541

[7] Rayner JJ Banerjee R Holloway CJ Lewis AJM Peterzan MA Francis JM. The relative contribution of metabolic and structural abnormalities to diastolic dysfunction in obesity. Int J Obes (Lond). (2018) 42(3):441–7. 10.1038/ijo.2017.239 28974742 PMC5880580

[8] Rucker D Joseph J. Defining the phenotypes for heart failure with preserved ejection fraction. Curr Heart Fail Rep. (2022) 19(6):445–57. 10.1007/s11897-022-00582-x 36178663

[9] Lewis GA Schelbert EB Williams SG Cunnington C Ahmed F McDonagh TA. Biological phenotypes of heart failure with preserved ejection fraction. J Am Coll Cardiol. (2017) 70(17):2186–200. 10.1016/j.jacc.2017.09.006 29050567

[10] Verma R Dhingra NK Connelly KA. Obesity/cardiometabolic phenotype of heart failure with preserved ejection fraction: mechanisms to recent trials. Curr Opin Cardiol. (2024) 39(2):92–7. 10.1097/HCO.0000000000001113 38294186

[11] Lam CSP Chandramouli C. Fat, female, fatigued: features of the obese HFpEF phenotype. JACC Heart Fail. (2018) 6(8):710–3. 10.1016/j.jchf.2018.06.006 30078394

[12] Tsutsui H Kinugawa S Matsushima S. Oxidative stress and heart failure. Am J Physiol Heart Circ Physiol. (2011) 301(6):H2181–90. 10.1152/ajpheart.00554.2011 21949114

[13] Gunes MF Akpinar MB Comertoglu I Akyol S Demircelik B Gurel OM. The investigation of a disintegrin and metalloproteinase with ThromboSpondin motifs (ADAMTS) 1, 5 and 16 in thoracic aortic aneurysms and dissections. Clin Lab. (2016) 62(3):425–33. 10.7754/Clin.Lab.2015.150730 27156333

[14] Zhazykbayeva S Pabel S Mugge A Sossalla S Hamdani N. The molecular mechanisms associated with the physiological responses to inflammation and oxidative stress in cardiovascular diseases. Biophys Rev. (2020) 12(4):947–68. 10.1007/s12551-020-00742-0 32691301 PMC7429613

[15] Ince H Kandemir E Bagci C Gulec M Akyol O. The effect of caffeic acid phenethyl ester on short-term acute myocardial ischemia. Med Sci Monit. (2006) 12(5):BR187–93. PMID: 16641873 16641873

[16] Budde H Hassoun R Mugge A Kovacs A Hamdani N. Current understanding of molecular pathophysiology of heart failure with preserved ejection fraction. Front Physiol. (2022) 13:928232. 10.3389/fphys.2022.928232 35874547 PMC9301384

[17] Paulus WJ Tschope C. A novel paradigm for heart failure with preserved ejection fraction: comorbidities drive myocardial dysfunction and remodeling through coronary microvascular endothelial inflammation. J Am Coll Cardiol. (2013) 62(4):263–71. 10.1016/j.jacc.2013.02.092 23684677

[18] van Heerebeek L Hamdani N Falcão-Pires I Leite-Moreira AF Begieneman MPV Bronzwaer JGF. Low myocardial protein kinase G activity in heart failure with preserved ejection fraction. Circulation. (2012) 126(7):830–9. 10.1161/CIRCULATIONAHA.111.076075 22806632

[19] Gevaert AB Boen JRA Segers VF Van Craenenbroeck EM. Heart failure with preserved ejection fraction: a review of cardiac and noncardiac pathophysiology. Front Physiol. (2019) 10:638. 10.3389/fphys.2019.00638 31191343 PMC6548802

[20] Stoicescu L Crişan D Morgovan C Avram L Ghibu S. Heart failure with preserved ejection fraction: the pathophysiological mechanisms behind the clinical phenotypes and the therapeutic approach. Int J Mol Sci. (2024) 25(2):794. 10.3390/ijms25020794 38255869 PMC10815792

[21] Brutsaert DL Meulemans AL Sipido KR Sys SU. Effects of damaging the endocardial surface on the mechanical performance of isolated cardiac muscle. Circ Res. (1988) 62(2):358–66. 10.1161/01.res.62.2.358 3338120

[22] Brutsaert DL. Cardiac endothelial-myocardial signaling: its role in cardiac growth, contractile performance, and rhythmicity. Physiol Rev. (2003) 83(1):59–115. 10.1152/physrev.00017.2002 12506127

[23] Lim SL Lam CS Segers VF Brutsaert DL De Keulenaer GW. Cardiac endothelium-myocyte interaction: clinical opportunities for new heart failure therapies regardless of ejection fraction. Eur Heart J. (2015) 36(31):2050–60. 10.1093/eurheartj/ehv132 25911648

[24] Segers VFM Brutsaert DL De Keulenaer GW. Cardiac remodeling: endothelial cells have more to say than just NO. Front Physiol. (2018) 9:382. 10.3389/fphys.2018.00382 29695980 PMC5904256

[25] Mohammed SF Hussain S Mirzoyev SA Edwards WD Maleszewski JJ Redfield MM. Coronary microvascular rarefaction and myocardial fibrosis in heart failure with preserved ejection fraction. Circulation. (2015) 131(6):550–9. 10.1161/CIRCULATIONAHA.114.009625 25552356 PMC4324362

[26] Akyildiz EU Celik S Ersoy G. Cardiac ruptures following myocardial infarction in medicolegal cases. Anadolu Kardiyol Derg. (2007) 7(3):253–6. PMID: 17785211 17785211

[27] Kendall RT Feghali-Bostwick CA. Fibroblasts in fibrosis: novel roles and mediators. Front Pharmacol. (2014) 5:123. 10.3389/fphar.2014.00123 24904424 PMC4034148

[28] Akyol O Akyol S Chen CH. Update on ADAMTS13 and VWF in cardiovascular and hematological disorders. Clin Chim Acta. (2016) 463:109–18. 10.1016/j.cca.2016.10.017 27746209

[29] Lopes Fernandes S Ribeiro Carvalho R Graça Santos L Montenegro Sá F Ruivo C Lázaro Mendes S. Pathophysiology and treatment of heart failure with preserved ejection fraction: state of the art and prospects for the future. Arq Bras Cardiol. (2020) 114(1):120–9. 10.36660/abc.20190111 31751442 PMC7025301

[30] Pehlivan S Gurses MS Ural MN Akyol S Eren F Turkmen Inanir N. The role of ADAMTS1 and versican in human myocardial infarction: a postmortem study. Lab Med. (2016) 47(3):205–12. 10.1093/labmed/lmw022 27346868 PMC4985771

[31] Regan TJ Lyons MM Ahmed SS Levinson GE Oldewurtel HA Ahmad MR. Evidence for cardiomyopathy in familial diabetes mellitus. J Clin Invest. (1977) 60(4):884–99. 10.1172/JCI108843 893679 PMC372437

[32] Muona P Peltonen J Jaakkola S Uitto J. Increased matrix gene expression by glucose in rat neural connective tissue cells in culture. Diabetes. (1991) 40(5):605–11. 10.2337/diab.40.5.605 2022305

[33] Da Dalt L Cabodevilla AG Goldberg IJ Norata GD. Cardiac lipid metabolism, mitochondrial function, and heart failure. Cardiovasc Res. (2023) 119(10):1905–14. 10.1093/cvr/cvad100 37392421 PMC10681665

[34] Lopaschuk GD Karwi QG Tian R Wende AR Abel ED. Cardiac energy metabolism in heart failure. Circ Res. (2021) 128(10):1487–513. 10.1161/CIRCRESAHA.121.318241 33983836 PMC8136750

[35] D'Souza K Nzirorera C Kienesberger PC. Lipid metabolism and signaling in cardiac lipotoxicity. Biochim Biophys Acta. (2016) 1861(10):1513–24. 10.1016/j.bbalip.2016.02.016 26924249

[36] Schulze PC. Myocardial lipid accumulation and lipotoxicity in heart failure. J Lipid Res. (2009) 50(11):2137–8. 10.1194/jlr.R001115 19687505 PMC2759818

[37] Henry JA Couch LS Rider OJ. Myocardial metabolism in heart failure with preserved ejection fraction. J Clin Med. (2024) 13(5):1195. 10.3390/jcm13051195 38592048 PMC10931709

[38] Goldberg IJ Trent CM Schulze PC. Lipid metabolism and toxicity in the heart. Cell Metab. (2012) 15(6):805–12. 10.1016/j.cmet.2012.04.006 22682221 PMC3387529

[39] Schulze PC Drosatos K Goldberg IJ. Lipid use and misuse by the heart. Circ Res. (2016) 118(11):1736–51. 10.1161/CIRCRESAHA.116.306842 27230639 PMC5340419

[40] Chen X Ruiz-Velasco A Zou Z Hille SS Ross C Fonseka O. PAK3 Exacerbates cardiac lipotoxicity via SREBP1c in obesity cardiomyopathy. Diabetes. (2024) 73(11):1805–20. 10.2337/db24-0240 39137120 PMC11493761

[41] Shimano H. SREBPs: physiology and pathophysiology of the SREBP family. FEBS J. (2009) 276(3):616–21. 10.1111/j.1742-4658.2008.06806.x 19143830

[42] Sack MN Rader TA Park S Bastin J McCune SA Kelly DP. Fatty acid oxidation enzyme gene expression is downregulated in the failing heart. Circulation. (1996) 94(11):2837–42. 10.1161/01.cir.94.11.2837 8941110

[43] Leggat J Bidault G Vidal-Puig A. Lipotoxicity: a driver of heart failure with preserved ejection fraction? Clin Sci (Lond). (2021) 135(19):2265–83. 10.1042/CS20210127 34643676 PMC8543140

[44] Lam CS Kraigher-Krainer DE Vasan E S R. Epidemiology and clinical course of heart failure with preserved ejection fraction. Eur J Heart Fail. (2011) 13(1):18–28. 10.1093/eurjhf/hfq121 20685685 PMC3003453

[45] Abel ED O'Shea KM Ramasamy R. Insulin resistance: metabolic mechanisms and consequences in the heart. Arterioscler Thromb Vasc Biol. (2012) 32(9):2068–76. 10.1161/ATVBAHA.111.241984 22895668 PMC3646067

[46] Roh J Hill JA Singh A Valero-Munoz M Sam F. Heart failure with preserved ejection fraction: heterogeneous syndrome, diverse preclinical models. Circ Res. (2022) 130(12):1906–25. 10.1161/CIRCRESAHA.122.320257 35679364 PMC10035274

[47] Capone F Sotomayor-Flores C Bode D Wang R Rodolico D Strocchi S. Cardiac metabolism in HFpEF: from fuel to signalling. Cardiovasc Res. (2023) 118(18):3556–75. 10.1093/cvr/cvac166 36504368

[48] El-Hafidi M Correa F Zazueta C. Mitochondrial dysfunction in metabolic and cardiovascular diseases associated with cardiolipin remodeling. Biochim Biophys Acta Mol Basis Dis. (2020) 1866(6):165744. 10.1016/j.bbadis.2020.165744 32105822

[49] Listenberger LL Han X Lewis SE Cases S Farese RV Ory DS. Triglyceride accumulation protects against fatty acid-induced lipotoxicity. Proc Natl Acad Sci U S A. (2003) 100(6):3077–82. 10.1073/pnas.0630588100 12629214 PMC152249

[50] Listenberger LL Schaffer JE. Mechanisms of lipoapoptosis: implications for human heart disease. Trends Cardiovasc Med. (2002) 12(3):134–8. 10.1016/s1050-1738(02)00152-4 12007739

[51] Adrian L Lenski M Todter K Heeren J Bohm M Laufs U. AMPK Prevents palmitic acid-induced apoptosis and lipid accumulation in cardiomyocytes. Lipids. (2017) 52(9):737–50. 10.1007/s11745-017-4285-7 28825205

[52] Wittenbecher C Eichelmann F Toledo E Guasch-Ferré M Ruiz-Canela M Li J. Lipid profiles and heart failure risk: results from two prospective studies. Circ Res. (2021) 128(3):309–20. 10.1161/CIRCRESAHA.120.317883 33272114 PMC7864881

[53] Law BA Liao X Moore KS Southard A Roddy P Ji R. Lipotoxic very-long-chain ceramides cause mitochondrial dysfunction, oxidative stress, and cell death in cardiomyocytes. FASEB J. (2018) 32(3):1403–16. 10.1096/fj.201700300R 29127192 PMC5892719

[54] Cornuault L Rouault P Duplaa C Couffinhal T Renault MA. Endothelial dysfunction in heart failure with preserved ejection fraction: what are the experimental proofs? Front Physiol. (2022) 13:906272. 10.3389/fphys.2022.906272 35874523 PMC9304560

[55] Karasawa T Takahashi M. Saturated fatty acid-crystals activate NLRP3 inflammasome. Aging (Albany NY). (2019) 11(6):1613–4. 10.18632/aging.101892 30923255 PMC6461185

[56] Batty M Bennett MR Yu E. The role of oxidative stress in atherosclerosis. Cells. (2022) 11(23):3843. 10.3390/cells11233843 36497101 PMC9735601

[57] Schiattarella GG Hill JA. Cardiometabolic HFpEF: mechanisms and therapies. Cardiometab Syndr J. (2021) 1(2):117–24. 10.51789/cmsj.2021.1.e18

[58] Peh ZH Dihoum A Hutton D Arthur JSC Rena G Khan F. Inflammation as a therapeutic target in heart failure with preserved ejection fraction. Front Cardiovasc Med. (2023) 10:1125687. 10.3389/fcvm.2023.1125687 37456816 PMC10339321

[59] Sun Q Güven B Wagg CS Almeida de Oliveira A Silver H Zhang L. Mitochondrial fatty acid oxidation is the major source of cardiac adenosine triphosphate production in heart failure with preserved ejection fraction. Cardiovasc Res. (2024) 120(4):360–71. 10.1093/cvr/cvae006 38193548

[60] Park T-S Hu Y Noh H-L Drosatos K Okajima K Buchanan J. Ceramide is a cardiotoxin in lipotoxic cardiomyopathy. J Lipid Res. (2008) 49(10):2101–12. 10.1194/jlr.M800147-JLR200 18515784 PMC2533410

[61] Lafuse WP Wozniak DJ Rajaram MVS. role of cardiac macrophages on cardiac inflammation, fibrosis and tissue repair. Cells. (2020) 10(1):51. 10.3390/cells10010051 33396359 PMC7824389

[62] Rosca MG Hoppel CL. Mitochondrial dysfunction in heart failure. Heart Fail Rev. (2013) 18(5):607–22. 10.1007/s10741-012-9340-0 22948484 PMC3855291

[63] Schiattarella GG Hill JA. Inhibition of hypertrophy is a good therapeutic strategy in ventricular pressure overload. Circulation. (2015) 131(16):1435–47. 10.1161/CIRCULATIONAHA.115.013894 25901069 PMC4408778

[64] He X Du T Long T Liao X Dong Y Huang ZP. Signaling cascades in the failing heart and emerging therapeutic strategies. Signal Transduct Target Ther. (2022) 7(1):134. 10.1038/s41392-022-00972-6 35461308 PMC9035186

[65] Zhao M Wang L Wang M Zhou S Lu Y Cui H. Targeting fibrosis, mechanisms and cilinical trials. Signal Transduct Target Ther. (2022) 7(1):206. 10.1038/s41392-022-01070-3 35773269 PMC9247101

[66] Sharma K Kass DA. Heart failure with preserved ejection fraction: mechanisms, clinical features, and therapies. Circ Res. (2014) 115(1):79–96. 10.1161/CIRCRESAHA.115.302922 24951759 PMC4146618

[67] Biernacka A Frangogiannis NG. Aging and cardiac fibrosis. Aging Dis. (2011) 2(2):158–73. PMID: 21837283 21837283 PMC3153299

[68] Schirone L Forte M Palmerio S Yee D Nocella C Angelini F. A review of the molecular mechanisms underlying the development and progression of cardiac remodeling. Oxid Med Cell Longev. (2017) 2017:3920195. 10.1155/2017/3920195 28751931 PMC5511646

[69] Briston T Selwood DL Szabadkai G Duchen MR. Mitochondrial permeability transition: a molecular lesion with multiple drug targets. Trends Pharmacol Sci. (2019) 40(1):50–70. 10.1016/j.tips.2018.11.004 30527591

[70] Singh RM Cummings E Pantos C Singh J. Protein kinase C and cardiac dysfunction: a review. Heart Fail Rev. (2017) 22(6):843–59. 10.1007/s10741-017-9634-3 28702857 PMC5635086

[71] Doenst T Nguyen TD Abel ED. Cardiac metabolism in heart failure: implications beyond ATP production. Circ Res. (2013) 113(6):709–24. 10.1161/CIRCRESAHA.113.300376 23989714 PMC3896379

[72] Daou D Gillette TG Hill JA. Inflammatory mechanisms in heart failure with preserved ejection fraction. Physiology (Bethesda). (2023) 38(5):217–230. 10.1152/physiol.00004.2023 PMC10396273 37013947

[73] Norata GD Caligiuri G Chavakis T Matarese G Netea MG Nicoletti A. The cellular and molecular basis of translational immunometabolism. Immunity. (2015) 43(3):421–34. 10.1016/j.immuni.2015.08.023 26377896

[74] Murphy MP O'Neill LAJ. Krebs cycle reimagined: the emerging roles of succinate and itaconate as signal transducers. Cell. (2018) 174(4):780–4. 10.1016/j.cell.2018.07.030 30096309

[75] Li C Qin D Hu J Yang Y Hu D Yu B. Inflamed adipose tissue: a culprit underlying obesity and heart failure with preserved ejection fraction. Front Immunol. (2022) 13:947147. 10.3389/fimmu.2022.947147 36483560 PMC9723346

[76] Gruzdeva OV Akbasheva OE Dyleva YA Antonova LV Matveeva VG Uchasova EG. Adipokine and cytokine profiles of epicardial and subcutaneous adipose tissue in patients with coronary heart disease. Bull Exp Biol Med. (2017) 163(5):608–11. 10.1007/s10517-017-3860-5 28948552

[77] Schiattarella GG Rodolico D Hill JA. Metabolic inflammation in heart failure with preserved ejection fraction. Cardiovasc Res. (2021) 117(2):423–34. 10.1093/cvr/cvaa217 32666082 PMC8599724

[78] Akyol O Chowdhury I Akyol HR Tessier K Vural H Akyol S. Why are cardiovascular diseases more common among patients with severe mental illness? The potential involvement of electronegative low-density lipoprotein (LDL) L5. Med Hypotheses. (2020) 142:109821. 10.1016/j.mehy.2020.109821 32417641

[79] Bancells C Canals F Benítez S Colomé N Julve J Ordóñez-Llanos J. Proteomic analysis of electronegative low-density lipoprotein. J Lipid Res. (2010) 51(12):3508–15. 10.1194/jlr.M009258 20699421 PMC2975723

[80] Ke L-Y Law SH Mishra VK Parveen F Chan H-C Lu Y-H. Molecular and cellular mechanisms of electronegative lipoproteins in cardiovascular diseases. Biomedicines. (2020) 8(12):550. 10.3390/biomedicines8120550 33260304 PMC7760527

[81] Chu C-S Law SH Lenzen D Tan Y-H Weng S-F Ito E. Clinical significance of electronegative low-density lipoprotein cholesterol in atherothrombosis. Biomedicines. (2020) 8(8):254. 10.3390/biomedicines8080254 32751498 PMC7460408

[82] Chen HH Hosken BD Huang M Gaubatz JW Myers CL Macfarlane RD. Electronegative LDLs from familial hypercholesterolemic patients are physicochemically heterogeneous but uniformly proapoptotic. J Lipid Res. (2007) 48(1):177–84. 10.1194/jlr.M500481-JLR200 17015886

[83] Chen C-H Jiang T Yang J-H Jiang W Lu J Marathe GK. Low-density lipoprotein in hypercholesterolemic human plasma induces vascular endothelial cell apoptosis by inhibiting fibroblast growth factor 2 transcription. Circulation. (2003) 107(16):2102–8. 10.1161/01.CIR.0000065220.70220.F7 12695302

[84] Yang CY Raya JL Chen H-H Chen C-H Abe Y Pownall HJ. Isolation, characterization, and functional assessment of oxidatively modified subfractions of circulating low-density lipoproteins. Arterioscler Thromb Vasc Biol. (2003) 23(6):1083–90. 10.1161/01.ATV.0000071350.78872.C4 12689919

[85] Ke L-Y Chan H-C Chen C-C Chang C-F Lu P-L Chu C-S. Increased APOE glycosylation plays a key role in the atherogenicity of L5 low-density lipoprotein. FASEB J. (2020) 34(7):9802–13. 10.1096/fj.202000659R 32501643

[86] Lee HC Akhmedov A Chen CH. Spotlight on very-low-density lipoprotein as a driver of cardiometabolic disorders: implications for disease progression and mechanistic insights. Front Cardiovasc Med. (2022) 9:993633. 10.3389/fcvm.2022.993633 36267630 PMC9577298

[87] Lin Y-S Liu C-K Lee H-C Chou M-C Ke L-Y Chen C-H. Electronegative very-low-density lipoprotein induces brain inflammation and cognitive dysfunction in mice. Sci Rep. (2021) 11(1):6013. 10.1038/s41598-021-85502-0 33727609 PMC7966811

[88] Shen M-Y Hsu J-F Chen F-Y Lu J Chang C-M Madjid M. Combined LDL and VLDL electronegativity correlates with coronary heart disease risk in asymptomatic individuals. J Clin Med. (2019) 8(8):1193. 10.3390/jcm8081193 31404961 PMC6723521

[89] Li C-L Chu C-H Lee H-C Chou M-C Liu C-K Chen C-H. Immunoregulatory effects of very low density lipoprotein from healthy individuals and metabolic syndrome patients on glial cells. Immunobiology. (2019) 224(5):632–7. 10.1016/j.imbio.2019.07.005 31402151

[90] Lee H-C Chen C-C Tsai W-C Lin H-T Shiao Y-L Sheu S-H. Very-Low-Density lipoprotein of metabolic syndrome modulates gap junctions and slows cardiac conduction. Sci Rep. (2017) 7(1):12050. 10.1038/s41598-017-11416-5 28935953 PMC5608762

[91] Lu C-W Lo Y-H Chen C-H Lin C-Y Tsai C-H Chen P-J. VLDL And LDL, but not HDL, promote breast cancer cell proliferation, metastasis and angiogenesis. Cancer Lett. (2017) 388:130–8. 10.1016/j.canlet.2016.11.033 27940127

[92] Lee H-C Lin H-T Ke L-Y Wei C Hsiao Y-L Chu C-S. VLDL From metabolic syndrome individuals enhanced lipid accumulation in atria with association of susceptibility to atrial fibrillation. Int J Mol Sci. (2016) 17(1):134. 10.3390/ijms17010134 26805814 PMC4730373

[93] Chen C-H Lu J Chen S-H Huang RY Yilmaz HR Dong J. Effects of electronegative VLDL on endothelium damage in metabolic syndrome. Diabetes Care. (2012) 35(3):648–53. 10.2337/dc11-1623 22279032 PMC3322679

[94] Estruch M Sanchez-Quesada JL Ordonez-Llanos J Benitez S. Inflammatory intracellular pathways activated by electronegative LDL in monocytes. Biochim Biophys Acta. (2016) 1861(9 Pt A):963–9. 10.1016/j.bbalip.2016.05.010 27235719

[95] Ohinata H Obama T Makiyama T Watanabe Y Itabe H. High-Density lipoprotein suppresses neutrophil extracellular traps enhanced by oxidized low-density lipoprotein or oxidized phospholipids. Int J Mol Sci. (2022) 23(22):13992. 10.3390/ijms232213992 36430470 PMC9698465

[96] Ren J Li H-W Chen L Zhang M Liu Y-X Zhang B-W. Mass spectrometry imaging-based single-cell lipidomics profiles metabolic signatures of heart failure. Research (Wash D C). (2023) 6:0019. 10.34133/research.0019 37040505 PMC10076023

[97] Du L Wang X Guo Y Tao T Wu H Xu X. Altered lipid metabolism promoting cardiac fibrosis is mediated by CD34(+) cell-derived FABP4(+) fibroblasts. Exp Mol Med. (2024) 56(8):1869–86. 10.1038/s12276-024-01309-9 39198543 PMC11372182

[98] Javaheri A Allegood JC Cowart LA Chirinos JA. Circulating ceramide 16:0 in heart failure with preserved ejection fraction. J Am Coll Cardiol. (2020) 75(17):2273–5. 10.1016/j.jacc.2020.02.062 32354389 PMC7375158

[99] Anker SD Usman MS Anker MS Butler J Böhm M Abraham WT. Patient phenotype profiling in heart failure with preserved ejection fraction to guide therapeutic decision making. A scientific statement of the heart failure association, the European heart rhythm association of the European Society of Cardiology, and the European society of hypertension. Eur J Heart Fail. (2023) 25(7):936–55. 10.1002/ejhf.2894 37461163

[100] Surendran A Zhang H Stamenkovic A Ravandi A. Lipidomics and cardiovascular disease. Biochim Biophys Acta Mol Basis Dis. (2025) 1871(5):167806. 10.1016/j.bbadis.2025.167806 40122185

[101] Costantino S Mengozzi A Velagapudi S Mohammed SA Gorica E Akhmedov A. Treatment with recombinant Sirt1 rewires the cardiac lipidome and rescues diabetes-related metabolic cardiomyopathy. Cardiovasc Diabetol. (2023) 22(1):312. 10.1186/s12933-023-02057-2 37957697 PMC10644415

[102] Staels B Dallongeville J Auwerx J Schoonjans K Leitersdorf E Fruchart JC. Mechanism of action of fibrates on lipid and lipoprotein metabolism. Circulation. (1998) 98(19):2088–93. 10.1161/01.cir.98.19.2088 9808609

[103] Derosa G Maffioli P. Peroxisome proliferator-activated receptor-gamma (PPAR-gamma) agonists on glycemic control, lipid profile and cardiovascular risk. Curr Mol Pharmacol. (2012) 5(2):272–81. 10.2174/1874467211205020272 22122457

[104] L Wang CC Hess CN Hiatt WR Goldfine AB. Clinical update: cardiovascular disease in diabetes Mellitus: atherosclerotic cardiovascular disease and heart failure in type 2 diabetes Mellitus - mechanisms, management, and clinical considerations. Circulation. (2016) 133(24):2459–502. 10.1161/CIRCULATIONAHA.116.022194 27297342 PMC4910510

[105] Kessler EL Oerlemans M Van Den Hoogen P Yap C Sluijter JPG De Jager SCA. Immunomodulation in heart failure with preserved ejection fraction: current state and future perspectives. J Cardiovasc Transl Res. (2021) 14(1):63–74. 10.1007/s12265-020-10026-3 32444946 PMC7892675

[106] Varughese MS Nayak AU Jacob S. Fenofibrate therapy in reducing the progression of diabetic retinopathy: revisiting the FIELD and ACCORD-EYE studies through the LENS trial. Eye (Lond). (2025) 39(1):15–7. 10.1038/s41433-024-03410-9 39438742 PMC11733283

[107] Charbonnel B Dormandy J Erdmann E Massi-Benedetti M Skene A, Group PRS. The prospective pioglitazone clinical trial in macrovascular events (PROactive): can pioglitazone reduce cardiovascular events in diabetes? Study design and baseline characteristics of 5238 patients. Diabetes Care. (2004) 27(7):1647–53. 10.2337/diacare.27.7.1647 15220241

[108] Sasaki H Asanuma H Fujita M Takahama H Wakeno M Ito S. Metformin prevents progression of heart failure in dogs: role of AMP-activated protein kinase. Circulation. (2009) 119(19):2568–77. 10.1161/CIRCULATIONAHA.108.798561 19414638

[109] Tong D Schiattarella GG Jiang N Daou D Luo Y Link MS. Impaired AMP-activated protein kinase signaling in heart failure with preserved ejection fraction-associated atrial fibrillation. Circulation. (2022) 146(1):73–6. 10.1161/CIRCULATIONAHA.121.058301 35858165 PMC9586456

[110] Anker SD Butler J Filippatos G Ferreira JP Bocchi E Böhm M. Empagliflozin in heart failure with a preserved ejection fraction. N Engl J Med. (2021) 385(16):1451–61. 10.1056/NEJMoa2107038 34449189

[111] Solomon SD McMurray JJV Claggett B De Boer RA DeMets D Hernandez AF. Dapagliflozin in heart failure with mildly reduced or preserved ejection fraction. N Engl J Med. (2022) 387(12):1089–98. 10.1056/NEJMoa2206286 36027570

[112] Kosiborod MN Abildstrøm SZ Borlaug BA Butler J Rasmussen S Davies M. Semaglutide in patients with heart failure with preserved ejection fraction and obesity. N Engl J Med. (2023) 389(12):1069–84. 10.1056/NEJMoa2306963 37622681

[113] Manubolu VS Lakshmanan S Kinninger A Ahmad K Susarla S Seok HJ. Effect of semaglutide on epicardial adipose tissue in type 2 diabetes: insights from the STOP (semaglutide treatment effect on coronary atherosclerosis progression). Randomized Trial. J Am Coll Cardiol. (2024) 84(9):865–7. 10.1016/j.jacc.2024.05.065 39168573

[114] Gaborit B. Effect of semaglutide on epicardial adipogenesis and hiPSC-atrial cardiomyocytes new hope in targeting epicardial adipose tissue. JACC Basic Transl Sci. (2025) 10(9):101349. 10.1016/j.jacbts.2025.101349 40992826 PMC12539453

[115] Martins FF Marinho TS Cardoso LEM Barbosa-da-Silva S Souza-Mello V Aguila MB. Semaglutide (GLP-1 receptor agonist) stimulates browning on subcutaneous fat adipocytes and mitigates inflammation and endoplasmic reticulum stress in visceral fat adipocytes of obese mice. Cell Biochem Funct. (2022) 40(8):903–13. 10.1002/cbf.3751 36169111

[116] Techorueangwiwat C Kanitsoraphan C Hansrivijit P. Therapeutic implications of statins in heart failure with reduced ejection fraction and heart failure with preserved ejection fraction: a review of current literature. F1000Res. (2021) 10:16. 10.12688/f1000research.28254.1 36873456 PMC9982192

[117] Anderson JL May HT Le VT Muhlestein JB Horne BD Bair TL. Impact of statin therapy in heart failure patients: results of a large real-world experience. JACC Adv. (2023) 2(4):100385. 10.1016/j.jacadv.2023.100385 38938227 PMC11198218

[118] Hamouche R Summers SA Holland WL Navankasattusas S Drakos SG Tseliou E. The role of sphingolipids in heart failure. Eur Heart J Open. (2025) 5(3):oeaf035. 10.1093/ehjopen/oeaf035 40322641 PMC12046129

[119] Xin J Liu R Deng M Song Y Cui Y Gao J. CTRP9 Ameliorates heart failure with preserved ejection fraction by regulating lipid metabolism. J Transl Med. (2026) 24(1):228. 10.1186/s12967-025-07661-2 41501803 PMC12903334

[120] Murphy SP Ibrahim NE Januzzi JL Jr. Heart failure with reduced ejection fraction: a review. JAMA. (2020) 324(5):488–504. 10.1001/jama.2020.10262 32749493

[121] Estruch R Ros E Salas-Salvadó J Covas M-I Corella D Arós F. Primary prevention of cardiovascular disease with a Mediterranean diet supplemented with extra-virgin olive oil or nuts. N Engl J Med. (2018) 378(25):e34. 10.1056/NEJMoa1800389 29897866

[122] Kitzman DW Brubaker PH Morgan TM Stewart KP Little WC. Exercise training in older patients with heart failure and preserved ejection fraction: a randomized, controlled, single-blind trial. Circ Heart Fail. (2010) 3(6):659–67. 10.1161/CIRCHEARTFAILURE.110.958785 20852060 PMC3065299

[123] Obokata M Reddy YNV Pislaru SV Melenovsky V Borlaug BA. Evidence supporting the existence of a distinct obese phenotype of heart failure with preserved ejection fraction. Circulation. (2017) 136(1):6–19. 10.1161/CIRCULATIONAHA.116.026807 28381470 PMC5501170

[124] Lucas JA Zhang Y Li P Gong K Miller AP Hassan E. Inhibition of transforming growth factor-beta signaling induces left ventricular dilation and dysfunction in the pressure-overloaded heart. Am J Physiol Heart Circ Physiol. (2010) 298(2):H424–32. 10.1152/ajpheart.00529.2009 19933419 PMC2822586

[125] Zhang H Zhan Q Huang B Wang Y Wang X. AAV-mediated gene therapy: advancing cardiovascular disease treatment. Front Cardiovasc Med. (2022) 9:952755. 10.3389/fcvm.2022.952755 36061546 PMC9437345

[126] Chamberlain K Riyad JM Weber T. Cardiac gene therapy with adeno-associated virus-based vectors. Curr Opin Cardiol. (2017) 32(3):275–82. 10.1097/HCO.0000000000000386 28169951 PMC5544589

[127] Yang L Xiao X. Creation of a cardiotropic adeno-associated virus: the story of viral directed evolution. Virol J. (2013) 10:50. 10.1186/1743-422X-10-50 23394344 PMC3574030

[128] Prakoso D Tate M Blasio MJ Ritchie RH. Adeno-associated viral (AAV) vector-mediated therapeutics for diabetic cardiomyopathy - current and future perspectives. Clin Sci (Lond). (2021) 135(11):1369–87. 10.1042/CS20210052 34076247 PMC8187922

[129] Ji R Akashi H Drosatos K Liao X Jiang H Kennel PJ. Increased *de novo* ceramide synthesis and accumulation in failing myocardium. JCI Insight. (2017) 2(9):e82922. 10.1172/jci.insight.82922 28469091 PMC5414571

[130] Nguyen Q Tung LW Lin B Sivakumar R Sar F Singhera G. Spatial transcriptomics in human cardiac tissue. Int J Mol Sci. (2025) 26(3):995. 10.3390/ijms26030995 39940764 PMC11817049

[131] Araz K Fioretti F Ladak S Obaidan M Butler J Hameed A. Phenotype characterization of heart failure with preserved ejection fraction in medical device and surgical trials. ESC Heart Fail. (2025) 12(6):3878–98. 10.1002/ehf2.15401 40857067 PMC12719810

[132] Patel R Wadid M Makwana B Kumar A Khadke S Bhatti A. GLP-1 Receptor agonists among patients with overweight or obesity, diabetes, and HFpEF on SGLT2 inhibitors. JACC Heart Fail. (2024) 12(11):1814–26. 10.1016/j.jchf.2024.07.006 39207323

[133] Chan H-C Chan H-C Wang L-F Chan M-L Huang W-C Bender D. The lysophosphatidylcholine-HIF-1alpha axis enhances apolipoprotein E sialylation and promotes electronegative LDL accumulation. iScience. (2026) 29(6):116167. 10.1016/j.isci.2026.116167 42291274 PMC13264260

[134] Faulin TES Kazuma SM Tripodi GL Cavalcante MF Wakasuqui F Oliveira CLP. Proinflammatory action of a new electronegative low-density lipoprotein epitope. Biomolecules. (2019) 9(8):386. 10.3390/biom9080386 31434316 PMC6723646

[135] Akyol O Chiang H-H Burns AR Yang C-Y Woodside DG Sawamura T. LDL Atherogenicity determined by size, density, oxidation, apolipoprotein(a), and electronegativity: an updated review. Front Cardiovasc Med. (2025) 12:1649759. 10.3389/fcvm.2025.1649759 41210345 PMC12592080

[136] Chiang HH Akyol O Ouyang F Hochman-Mendez C Mesquita F Chen CH. Electronegative VLDL drives maturity-dependent cellular dysfunction on human cardiomyocytes. In: 2025 American Heart Association scientific sessions - meeting abstract (4368942). Circulation. (2025) 152 (Suppl_3):1. 10.1161/circ.152.suppl_3.4368942

[137] Chiang H-H Akyol O Tsai M-H Hochman C Vykoukal J Fahrmann J. Electronegative VLDL impairs mitochondrial function and promotes cardiomyocyte dysfunction in heart failure with preserved ejection fraction. In: 2024 American Heart Association scientific sessions - meeting abstract (4144929). Circulation. (2024) 150(Suppl_1):1. 10.1161/circ.150.suppl_1.4144929

[138] Klingenberg R Luscher TF. Inflammation in coronary artery disease and acute myocardial infarction - is the stage set for novel therapies? Curr Pharm Des. (2012) 18(28):4358–69. 10.2174/138161212802481219 22390645

[139] Chen C-H Sawamura T Akhmedov A Tsai M-H Akyol O Kakino A. Evolving concepts of low-density lipoprotein: from structure to function. Eur J Clin Invest. (2025) 55(5):e70019. 10.1111/eci.70019 40045739

[140] Ghosh AK Vaughan DE. PAI-1 in tissue fibrosis. J Cell Physiol. (2012) 227(2):493–507. 10.1002/jcp.22783 21465481 PMC3204398

[141] Borén J Chapman MJ Krauss RM Packard CJ Bentzon JF Binder CJ. Low-density lipoproteins cause atherosclerotic cardiovascular disease: pathophysiological, genetic, and therapeutic insights: a consensus statement from the European atherosclerosis society consensus panel. Eur Heart J. (2020) 41(24):2313–30. 10.1093/eurheartj/ehz962 32052833 PMC7308544

[142] Vural H Armutcu F Akyol O Weiskirchen R. The potential pathophysiological role of altered lipid metabolism and electronegative low-density lipoprotein (LDL) in non-alcoholic fatty liver disease and cardiovascular diseases. Clin Chim Acta. (2021) 523:374–9. 10.1016/j.cca.2021.10.018 34678296

[143] Ke LY Stancel N Bair H Chen CH. The underlying chemistry of electronegative LDL's Atherogenicity. Curr Atheroscler Rep. (2014) 16(8):428. 10.1007/s11883-014-0428-y 24890631

